# Supplementary material for: Online Module to Improve Emergency Department Observation Unit Practice
Source: MedEdPORTAL. 2016 Jul 8;12:10423. doi: 10.15766/mep_2374-8265.10423 (PMC6464449; doi:10.15766/mep_2374-8265.10423)
Supplement: Supplementary file 1 — A. Introducing Observation Medicine for Emergency Medicine Physicians Articulate folder B. Introducing Observation Medicine for Emergency Medicine Physicians PowerPoint.pptx C. Articulate Presentation Instructions.txt [file mep-12-10423-s001.zip › _Educational Summary Report_10423.pdf]

MedEdPORTAL®
The Journal of Teaching and Learning Resources

Crossref

Original Publication

OPEN ACCESS

Online Module to Improve Emergency Department Observation Unit Practice

Sangil Lee, MD*, Ian Young, MD, James Colletti, MD
*Corresponding author: Lee.Sangil@mayo.edu

Citation: Lee S, Young I, Colletti J. Online module to improve emergency department observation unit practice. *MedEdPORTAL*. 2016;12:10423. https://doi.org/10.15766/mep_2374-8265.10423

Copyright: © 2016 Lee et al. This is an open-access article distributed under the terms of the Creative Commons Attribution license.

Appendices

A. Introducing Observation Medicine for Emergency Medicine Physicians Articulate folder
B. Introducing Observation Medicine for Emergency Medicine Physicians PowerPoint.pptx
C. Articulate Presentation Instructions.txt

All appendices are peer reviewed as integral parts of the Original Publication.

Abstract

Introduction: The emergency department (ED) observation unit has become a unique practice opportunity to liberate inpatient capacity in the United States. Substantial variations in clinical practice and level of comfort among ED providers existed in a large health care system. We aimed to study the effectiveness of an educational module targeted for emergency medicine providers. **Methods:** We developed a 20-minute online module including pretest, learning module, and posttest. The format consisted of Likert-scale (1 = least comfortable, 5 = most comfortable), true-or-false, and multiple-choice questions. The learning module contained slides, script, and figures describing management strategy for commonly encountered conditions in the observation unit. The institutional review board granted exempted review. Pre- and posttest scores were evaluated with the Wilcoxon rank-sum test, and a p value of less than .05 was considered significant. **Results:** Twenty-one participants completed the pretest, with a mean score of 63.8 (SD = 19.3), and 14 participants completed the posttest, with a mean score of 87.9 (SD = 9.7; p = .0001). Eight participants responded to a follow-up survey, a response rate of 57%. Responses demonstrated that levels of provider comfort in selection of observation (M = 4.3, SD = 1.5), choice of stress test for chest pain (M = 4.3, SD = 1.0), asthma management (M = 4.1, SD = 1.0), anaphylaxis care (M = 4.1, SD = 1.4), and documentation (M = 4.3, SD = 0.9) were high. **Discussion:** An online learning module can be useful to enhance the knowledge of observation medicine among emergency medicine providers.

Keywords

Adult Learning, Online Learning Module, Observation Medicine, Practice Standardization

Educational Objectives

By the end of this module, the learner will be able to:

1. Demonstrate how emergency department patients are dispositioned to the observation unit after the initial assessment, evaluation, and management.
2. Evaluate diagnostic strategies for diagnoses that lend themselves to observation unit care in a case scenario.
3. Formulate the management strategies for core diagnosis in observation medicine (chest pain, renal colic, asthma, cellulitis, nausea/vomiting/diarrhea, and anaphylaxis).
4. Demonstrate the requirement for documentation, billing, and reimbursement as it relates to observation medicine.

Introduction

A change in the health care model for an aging population, the complexity of medical conditions, and hospital overcrowding have led to the pursuit of a cost-driven practice model. A short-stay unit within the emergency department (ED) emerged to become the ED observation unit.[1] The ED observation unit has been providing a unique practice opportunity to liberate inpatient capacity since the 1990s in the United States.[2] Several studies have reported its value as an alternative to hospitalization.[3-5]

Although observation medicine is a part of the requirement for the Model of the Clinical Practice of Emergency Medicine (EM Model),[6] not all the existing emergency medicine training sites have ED observation units,[7] resulting in variable learning experiences for trainees. Our health system launched an

ED observation unit in 2014. Substantial variations in clinical practice and levels of comfort among ED providers were observed when we launched the ED observation unit. Therefore, we found it necessary to fill the knowledge gaps we had identified in the practice of observation medicine.

Our study's aim was threefold:

1. To determine whether the introduction of an online instructional module enhanced ED providers' knowledge and retention of the practice of observation medicine.
2. To ascertain if an online instructional module improved ED providers' comfort level with care delivery in an observation unit.
3. To evaluate utilization of the ED observation unit in relation to admission rate.

Methods

We developed an online training session based on a review of existing literature and stakeholder input (academic emergency medicine physicians and cardiologists).[8-12] The online training session included a pretest, a learning module, and a posttest using an online platform. The pre- and posttest assessments consisted of a Likert scale (1 = least comfortable, 5 = most comfortable), true-or-false questions, and multiple-choice questions.

Before completing the observation medicine module (Appendix A or B; to open Appendix A, please follow the instructions given in Appendix C), the learner completed a survey embedded in the slide. The pretest survey consisted of two questions related to the background information of the learner as well as 10 questions designed to assess his or her baseline knowledge before module learning started. The multiple-question format and the Likert scale were used to assess the learner's level of training and experience in observation medicine. These were either true-or-false or multiple-choice questions where the learner was presented with a clinical scenario and asked to respond to demonstrate his or her understanding of observation medicine. The learning module contained slides, scripts, and figures describing inclusion, exclusion, and management strategies for commonly encountered conditions in the observation unit. Similar to the pretest, the participants were prompted to take a total of 10 questions assessing their understanding of observation medicine followed by two questions rating the level of comprehension. This self-assessment was administered through an online module. Pre- and postmodule assessments were conducted electronically. We invited participants by e-mail in February of 2015 and sent a reminder 2 weeks later. All participants were asked to participate voluntarily and remained anonymous.

The online platform Articulate Online was utilized to upload the assessment. The initial evaluation was followed by administration of the education module, and then, posttest evaluation was completed. This module was administered in the winter, with the goal of our providers having 6 months of exposure to observation unit practice. All pre- and posttest questions were accompanied by answers, and providers were given a score at the end of the test. Participants were asked to spend 20 minutes reviewing the observation unit overview presentation, observation unit inclusion and exclusion criteria, and documentation with a billing component as a part of the module. In order to take the module, participants were required to have institution employee ID and Internet access. Instructors were advised to include this module as a part of self-learning for new providers prior to rotating ED observation unit during orientation, and individual feedback with the case-based review was provided to retain or improve providers' comfort levels.

For validity, we applied consequence validity as our module was designed to improve providers' knowledge in the management of observation unit patients, retention of comfort level, and increased utilization in the observation unit. We also reported the total number of ED observation unit admissions with subsequent discharge, as well as the average length of stay in ED observation unit and inpatient observation unit during the year 2014. The study protocol was exempted by the institutional reviewer board at the Mayo Clinic.

Results

The online module was developed in February of 2015 and administered to a total of 21 participants consisting of emergency medicine physicians, resident physicians, and advance practice providers from the Mayo Clinic Health System in Mankato, Minnesota; Eau Claire, Wisconsin; and the Mayo Clinic in Rochester, Minnesota, by invitation. Currently, there is no other publication with our instrument. This instrument was tested in a site where observation units are managed by emergency medicine providers, and we did not include other Mayo Clinic sites that did not have ED observation units.

We reported consequence validity in three ways. First, this was a formative assessment to provide the instructional module between the pre- and posttests. A total of 21 participants completed the pretest, with a mean score of 63.8 ($SD = 19.3$), and a total of 14 participants completed the posttest, with a mean score of 87.9 ($SD = 9.7$), which was statistically significant ($p = .0001$). Second, a total of eight participants responded to a follow-up survey 2 months after module administration, for a response rate of 57%. Responses demonstrated that level of provider comfort in selection of observation ($M = 4.3$, $SD = 1.5$), choice of stress test for chest pain ($M = 4.3$, $SD = 1.0$), asthma management ($M = 4.1$, $SD = 1.0$), anaphylaxis care ($M = 4.1$, $SD = 1.4$), and documentation ($M = 4.3$, $SD = 0.9$) remained high. All of the respondents agreed that the module was helpful. We made the module available to learners who required additional review, and instructors continued to review individual cases and provide feedback to the individual or group. Third, the observation unit census after administration of the module was compared to the census before the module; it was analyzed by matched *t* test and showed a statistically significant increase throughout the period ($p = .0014$; see Figure). The overall admission rate from the ED observation unit remained less than 20% after implementation. During 2015, a total of 553 patients remained in the ED observation unit and were subsequently discharged. Furthermore, the overall length of stay in the ED observation unit averaged 10 hours, whereas the average length of stay in inpatient observation was 20 hours. These imply that our module led to improved utilization without negatively affecting the quality of care in the unit.

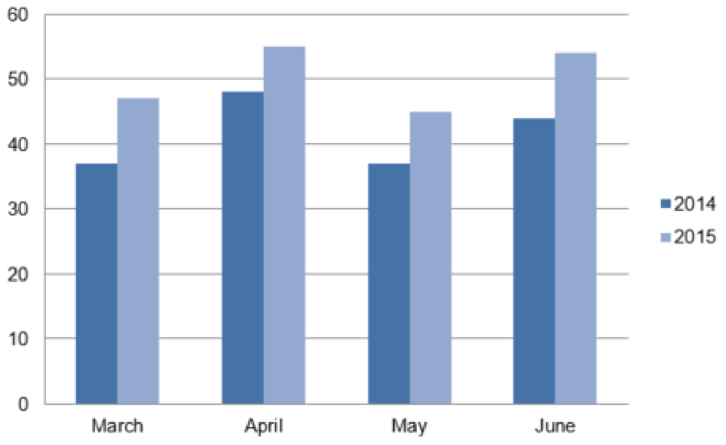

Figure 1. Emergency department observation unit census before (2014) and after (2015) module administration. $p = .0014$ (matched *t* test).

Discussion

We developed a 20-minute online module including pretest, learning content, and posttest to provide an educational resource to practicing emergency medicine providers within a large health care system. The results showed significant improvement at posttest, high comfort levels among providers who took the module, and an increase in a census of ED observation unit utilization.

This online module could be used with any level of learners in emergency medicine, including medical students as a part of an emergency medicine curriculum, resident physicians, and advanced practice

providers in the emergency medicine at the graduate medical education level, in addition to junior-level emergency medicine providers as a part of continuous medical education activities. Instructors can incorporate the module as a part of orientation, and as a benefit of asynchronous learning, the module can serve as a supplement to the learning activity at low fidelity.

This instrument has several limitations. First, the observation training module was primarily developed for practicing emergency medicine physicians; therefore, we need further validation by testing the instrument on resident physicians and advanced practice providers in training. Second, the response rate was approximately 57%. Thus, we were unable to determine how the nonresponse group would have performed (creating a concern for nonresponse bias). Third, this instrument was tested on clinical sites within the Mayo Clinic that share similar practice standards. It may not be generalizable to other health care sites. Our practice considered only renal colic among abdominal pain cases, as most of our providers did not think the undifferentiated abdominal pain would be a good candidate. We suggest the instrument be revised to suit your own institution.

Future opportunities include expanding the target audience to different institutions and including a similar number of participants at different training stages, as this would enable us to evaluate generalizability and add to the validity of evidence in internal structure. It would be interesting to see how much this module can cover observation medicine from the EM Model[6] and contribute to competency assessment in observation and reassessment—reevaluates patients undergoing ED observation (and monitoring) and, using appropriate data and resources, determines the differential diagnosis, treatment plan, and disposition—as specified by the emergency medicine milestone project.[13]

In summary, online learning modules can be useful to enhance the knowledge of observation medicine among emergency medicine providers. Currently, this learning module is integrated as a part of the education resources for new providers within our health care system.

Sangil Lee, MD: Emergency Medicine Physician, Mayo Clinic Health System, Mankato, Minnesota

Ian Young, MD: Urgent Care Physician, Winona Health in Winona, Minnesota

James Colletti, MD: Associate Professor of Emergency Medicine, Mayo Clinic

Disclosures
None to report.

Funding/Support
None to report.

Ethical Approval
This publication contains data obtained from human subjects and received ethical approval.

References

1. Hess EP, Nestler DM. Transforming the emergency department observation unit: a look into the future. *Cardiol Clin*. 2012;30(4):501-521. http://dx.doi.org/10.1016/j.ccl.2012.07.013
2. Graff LG, Dallara J, Ross MA, et al. Impact on the care of the emergency department chest pain patient from the chest pain evaluation registry (CHEPER) study. *Am J Cardiol*. 1997;80(5):563-568. http://dx.doi.org/10.1016/S0002-9149(97)00422-0
3. Rydman RJ, Isola ML, Roberts RR, et al. Emergency department observation unit versus hospital inpatient care for a chronic asthmatic population: a randomized trial of health status outcome and cost. *Med Care*. 1998;36(4):599-609. http://dx.doi.org/10.1097/00005650-199804000-00015
4. Ross MA, Compton S, Richardson D, Jones R, Nittis T, Wilson A. The use and effectiveness of an emergency department observation unit for elderly patients. *Ann Emerg Med*. 2003;41(5):668-677. http://dx.doi.org/10.1067/mem.2003.153
5. Baugh CW, Liang L-J, Probst MA, Sun BC. National cost savings from observation unit management of syncope.*Acad Emerg Med*. 2015;22(8):934-941. http://dx.doi.org/10.1111/acem.12720

6. Counselman FL, Borenstein MA, Chisholm CD, et al. The 2013 Model of the Clinical Practice of Emergency Medicine.*Acad Emerg Med*. 2014;21(5):574-598. http://dx.doi.org/10.1111/acem.12373
7. Yealy DM, De Hart DA, Ellis G, Wolfson AB. A survey of observation units in the United States.*Am J Emerg Med*. 1989;7(6):576-580. http://dx.doi.org/10.1016/0735-6757(89)90277-5
8. Rydman RJ, Zalenski RJ, Roberts RR, et al. Patient satisfaction with an emergency department chest pain observation unit.*Ann Emerg Med*. 1997;29(1):109-115. http://dx.doi.org/10.1016/S0196-0644(97)70316-0
9. Venkatesh AK, Geisler BP, Gibson Chambers JJ, Baugh CW, Bohan JS, Schuur JD. Use of observation care in US emergency departments, 2001 to 2008. *PloS One*. 2011;6(9):e24326. http://dx.doi.org/10.1371/journal.pone.0024326
10. Amsterdam EA, Kirk JD, Bluemke DA, et al. Testing of low-risk patients presenting to the emergency department with chest pain: a scientific statement from the American Heart Association. *Circulation*. 2010;122(17):1756-1776. http://dx.doi.org/10.1161/CIR.0b013e3181ec61df
11. Campbell RL, Li JT, Nicklas RA, Sadosty AT. Emergency department diagnosis and treatment of anaphylaxis: a practice parameter. *Ann Allergy Asthma Imnunol*. 2014;113(6):599-608. http://dx.doi.org/10.1016/j.anai.2014.10.007
12. Baugh CW, Venkatesh AK, Hilton JA, Samuel PA, Schuur JD, Bohan JS. Making greater use of dedicated hospital observation units for many short-stay patients could save $3.1 billion a year. *Health Aff (Millwood)*. 2012;31(10):2314-2323. http://dx.doi.org/10.1377/hlthaff.2011.0926
13. Beeson MS, Carter WA, Christopher TA, et al. The development of the emergency medicine milestones.*Acad Emerg Med*. 2013;20(7):724-729. http://dx.doi.org/10.1111/acem.12157

Received: October 1, 2015 | **Accepted:** May 18, 2016 | **Published:** July 8, 2016
