# Supplementary material for: Online Module to Improve Emergency Department Observation Unit Practice
Source: MedEdPORTAL. 2016 Jul 8;12:10423. doi: 10.15766/mep_2374-8265.10423 (PMC6464449; doi:10.15766/mep_2374-8265.10423)
Supplement: Supplementary file 1 — A. Introducing Observation Medicine for Emergency Medicine Physicians Articulate folder B. Introducing Observation Medicine for Emergency Medicine Physicians PowerPoint.pptx C. Articulate Presentation Instructions.txt [file mep-12-10423-s001.zip › A. Introducing Observation Medicine for Emergency Medicine Physicians Articulate/mobile/mobileinstall.html]

4\_8\_2016\_Instructional\_module\_after


# Get the free Articulate Mobile Player app

You'll need it to view your course on your iPad. Just download it from the App Store.

Available on the App Store
