# Supplementary material for: Online Module to Improve Emergency Department Observation Unit Practice
Source: MedEdPORTAL. 2016 Jul 8;12:10423. doi: 10.15766/mep_2374-8265.10423 (PMC6464449; doi:10.15766/mep_2374-8265.10423)
Supplement: Supplementary file 1 — A. Introducing Observation Medicine for Emergency Medicine Physicians Articulate folder B. Introducing Observation Medicine for Emergency Medicine Physicians PowerPoint.pptx C. Articulate Presentation Instructions.txt [file mep-12-10423-s001.zip › B. Introducing Observation Medicine for Emergency Medicine Physicians PowerPoint.pptx]

## Slide 1
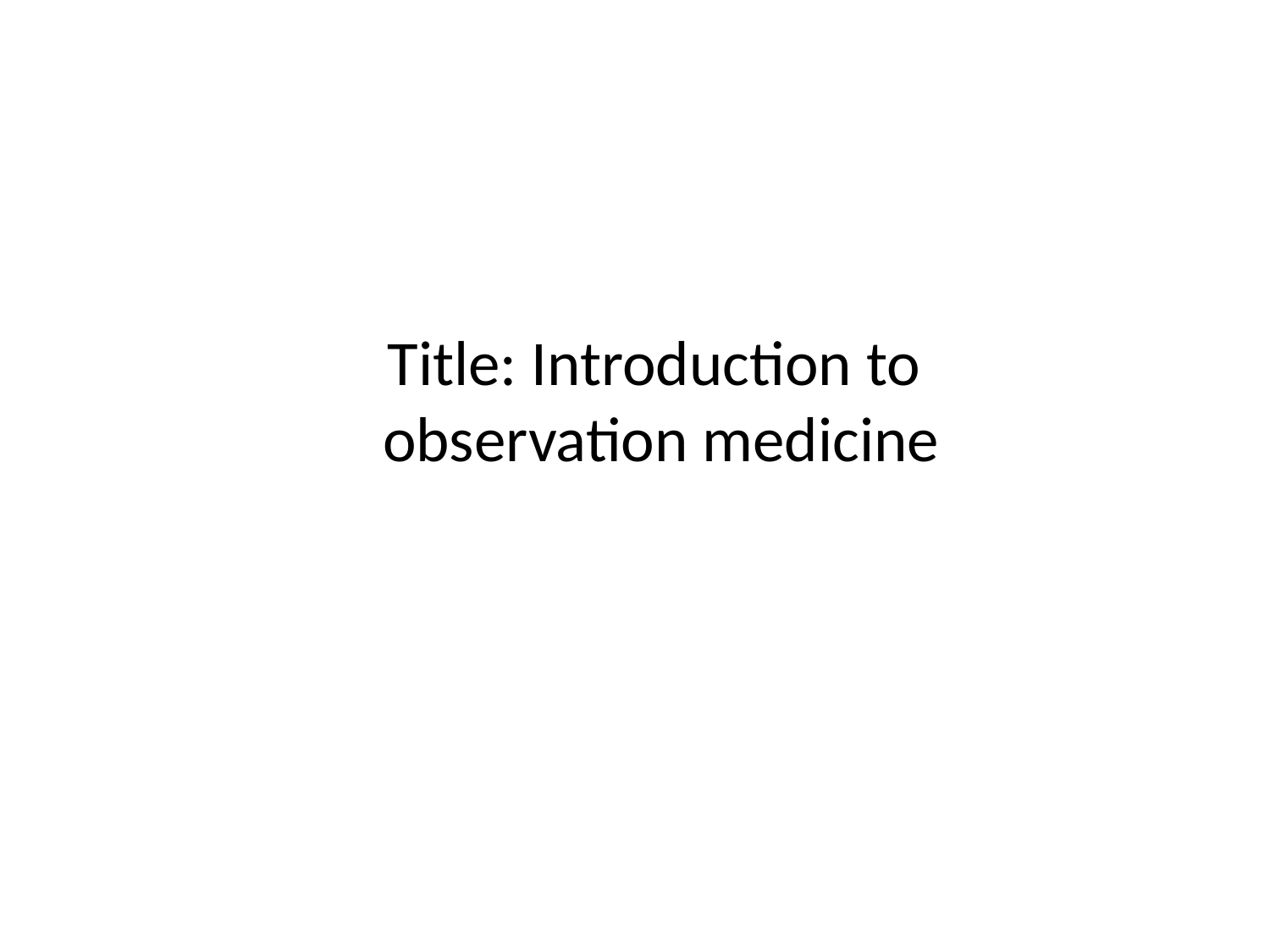

# Title: Introduction to observation medicine

## Slide 2
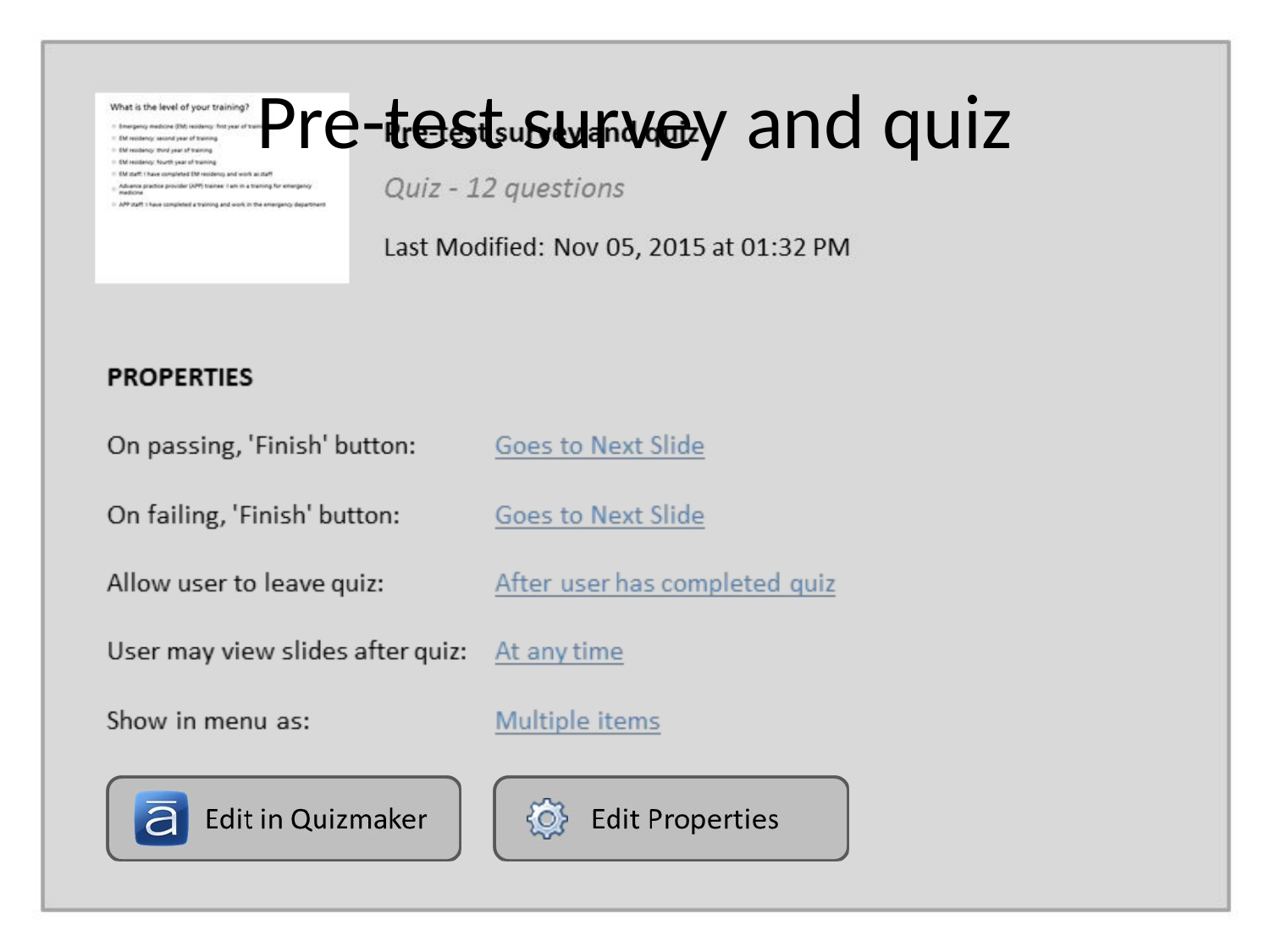

# Pre-test survey and quiz

## Slide 3
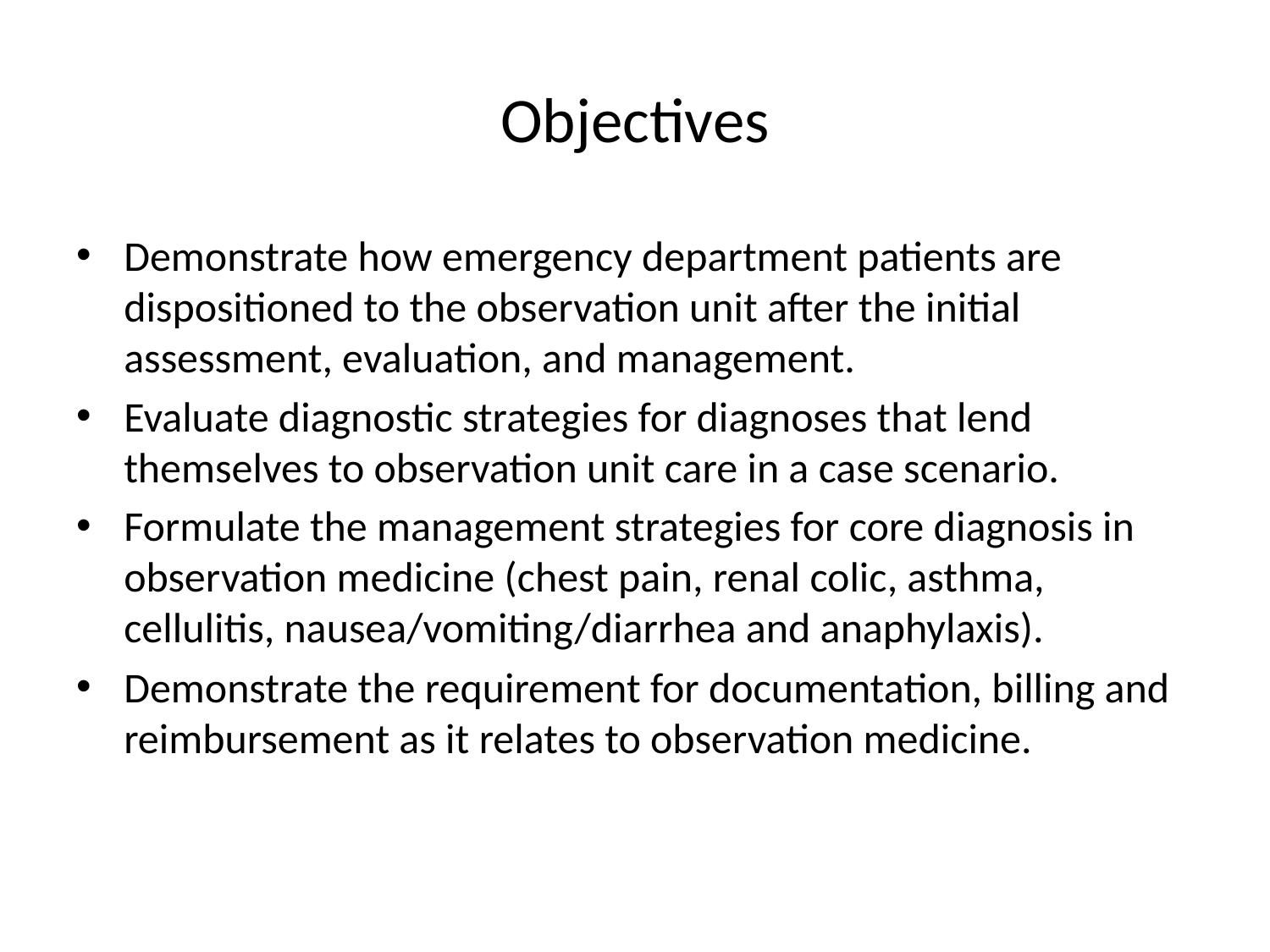

# Objectives
Demonstrate how emergency department patients are dispositioned to the observation unit after the initial assessment, evaluation, and management.
Evaluate diagnostic strategies for diagnoses that lend themselves to observation unit care in a case scenario.
Formulate the management strategies for core diagnosis in observation medicine (chest pain, renal colic, asthma, cellulitis, nausea/vomiting/diarrhea and anaphylaxis).
Demonstrate the requirement for documentation, billing and reimbursement as it relates to observation medicine.

## Slide 4
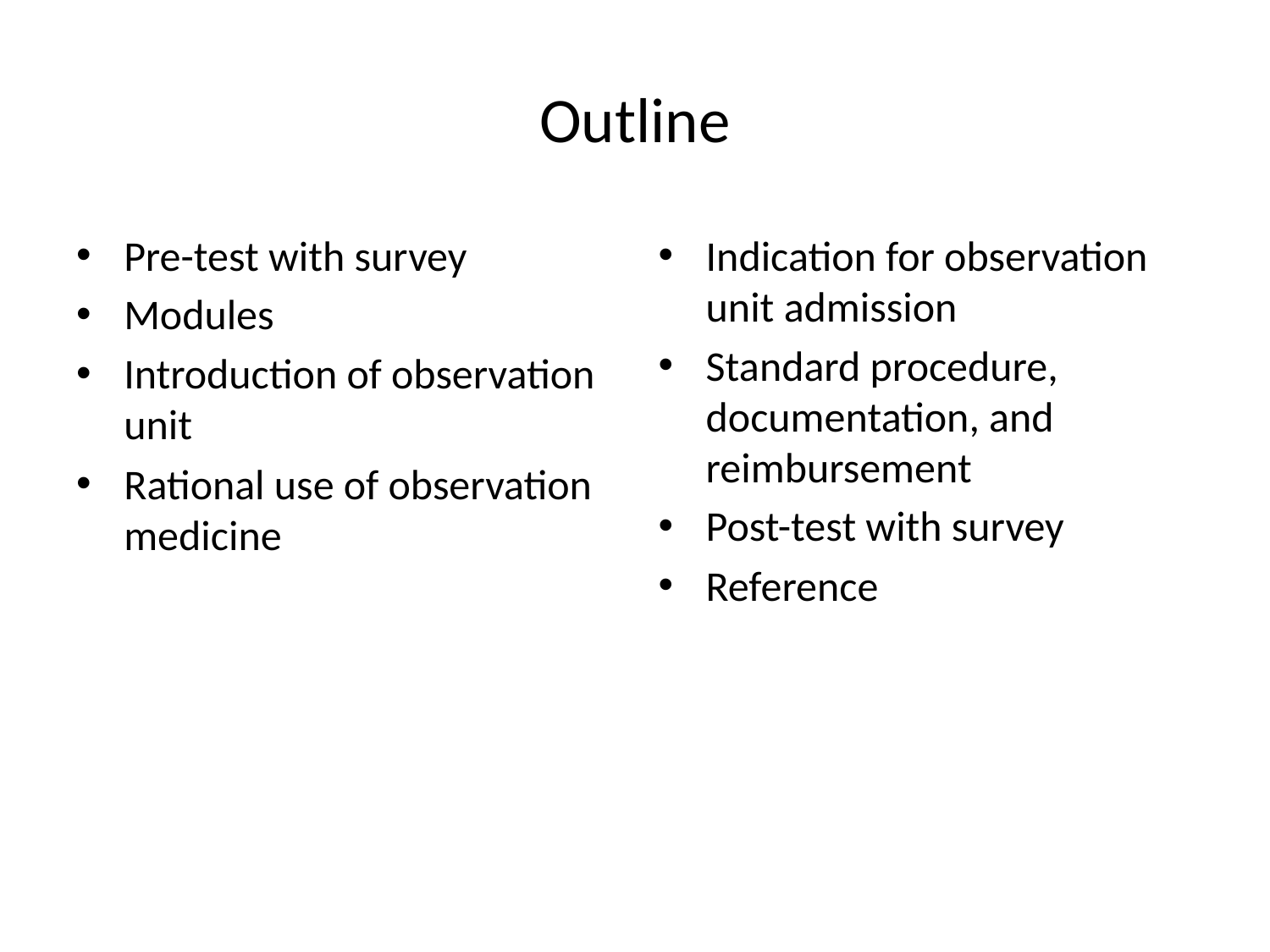

# Outline
Pre-test with survey
Modules
Introduction of observation unit
Rational use of observation medicine
Indication for observation unit admission
Standard procedure, documentation, and reimbursement
Post-test with survey
Reference

## Slide 5
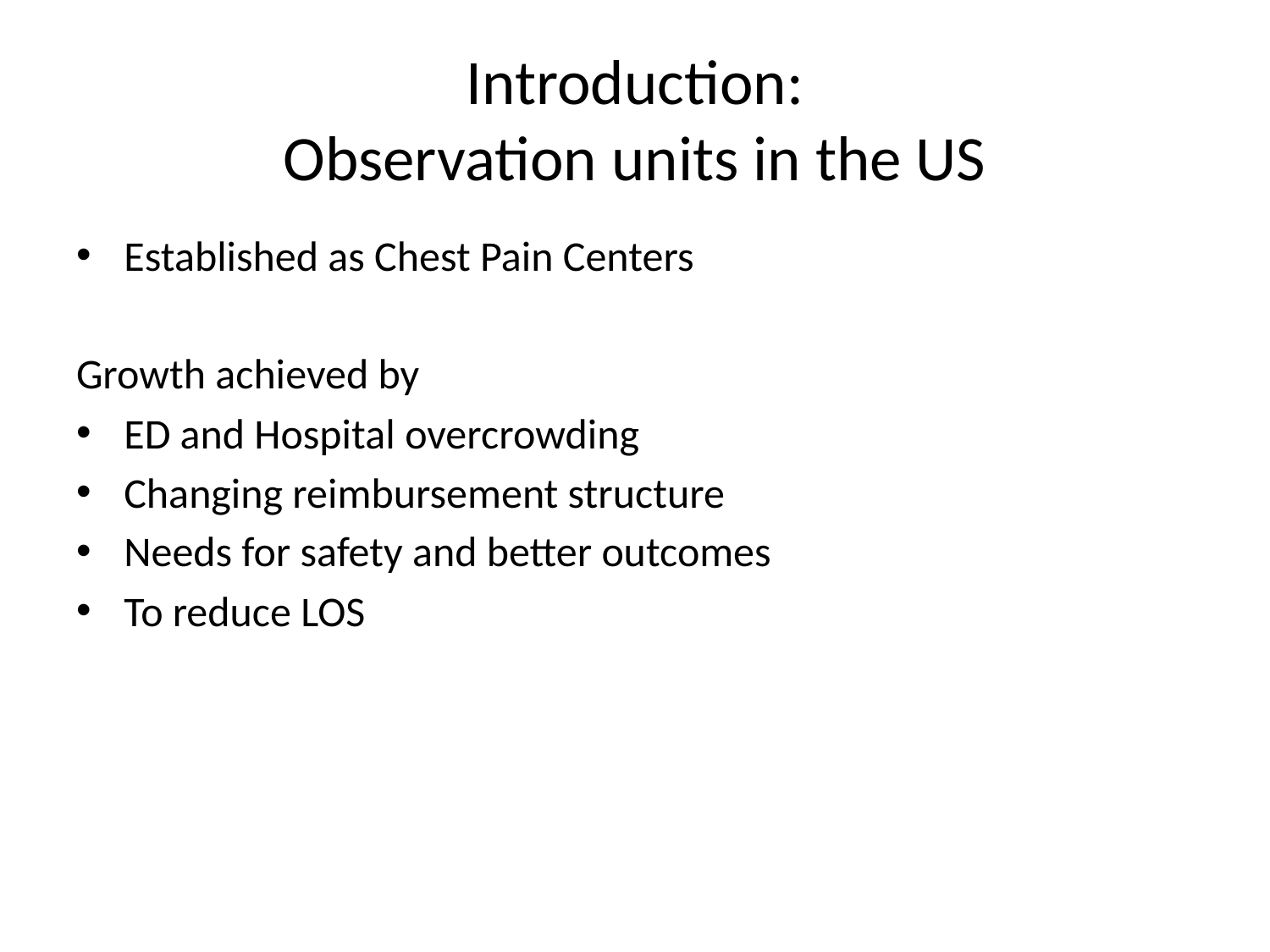

# Introduction: Observation units in the US
Established as Chest Pain Centers
Growth achieved by
ED and Hospital overcrowding
Changing reimbursement structure
Needs for safety and better outcomes
To reduce LOS

## Slide 6
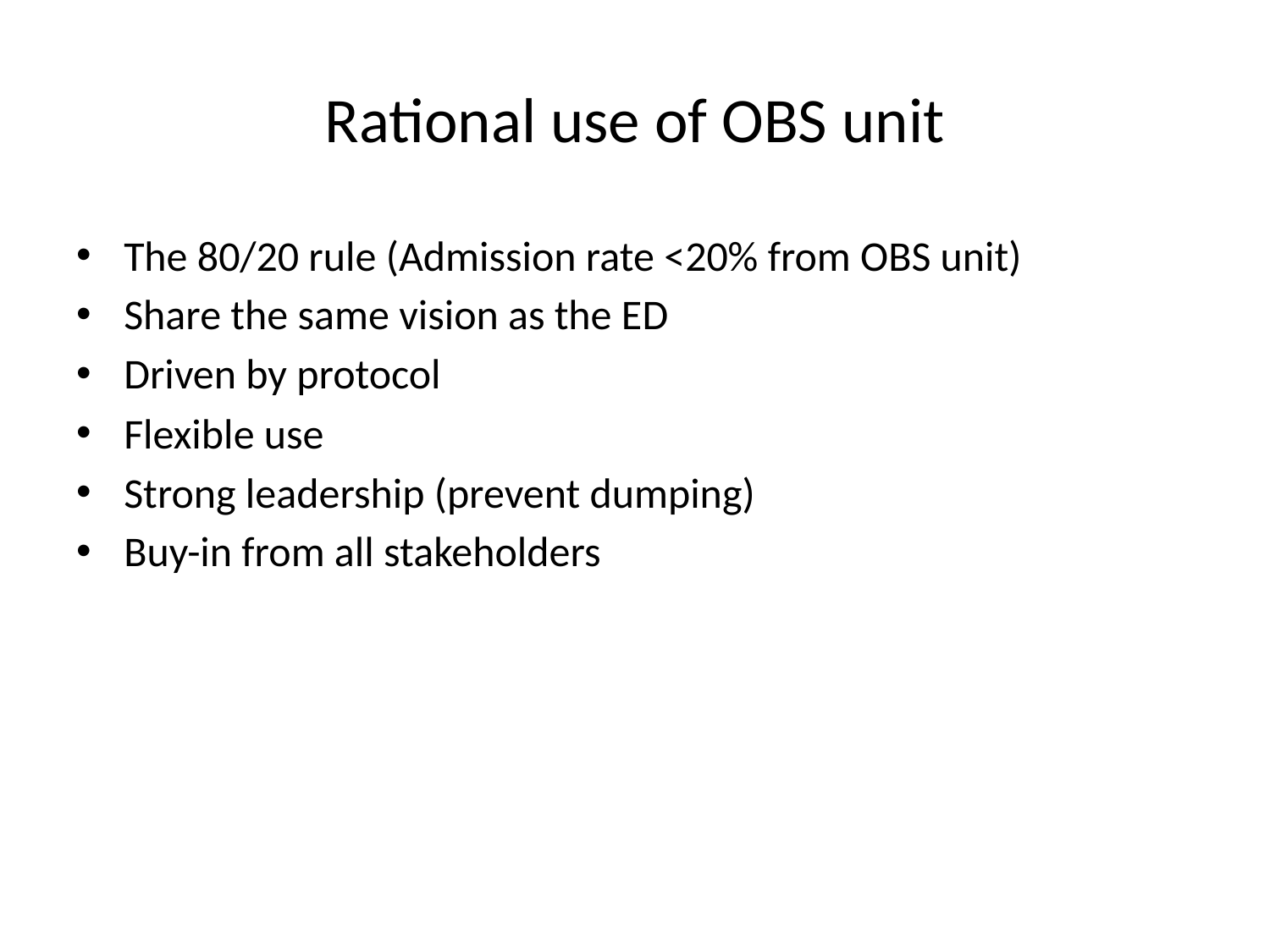

# Rational use of OBS unit
The 80/20 rule (Admission rate <20% from OBS unit)
Share the same vision as the ED
Driven by protocol
Flexible use
Strong leadership (prevent dumping)
Buy-in from all stakeholders

## Slide 7
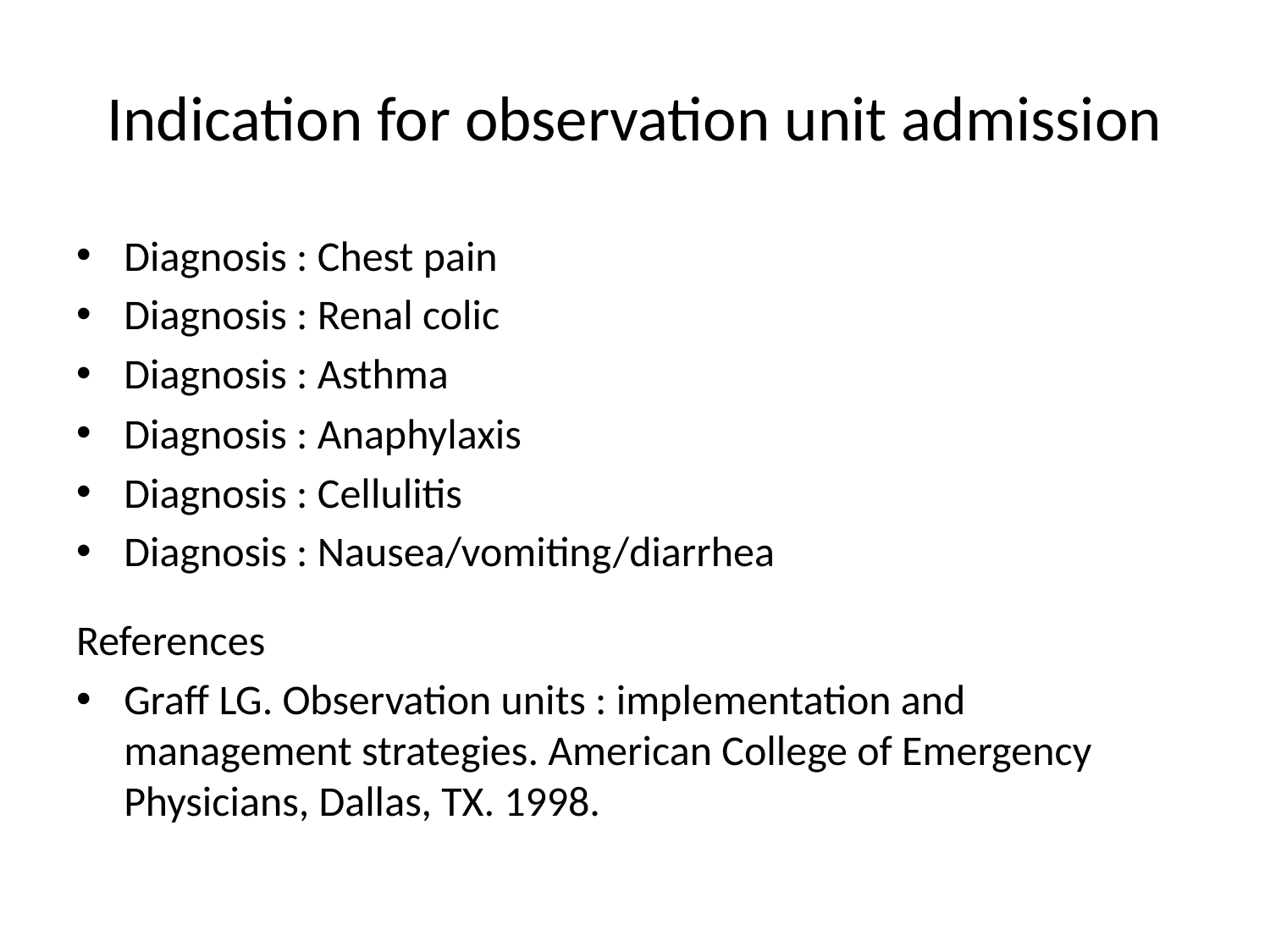

# Indication for observation unit admission
Diagnosis : Chest pain
Diagnosis : Renal colic
Diagnosis : Asthma
Diagnosis : Anaphylaxis
Diagnosis : Cellulitis
Diagnosis : Nausea/vomiting/diarrhea
References
Graff LG. Observation units : implementation and management strategies. American College of Emergency Physicians, Dallas, TX. 1998.

## Slide 8
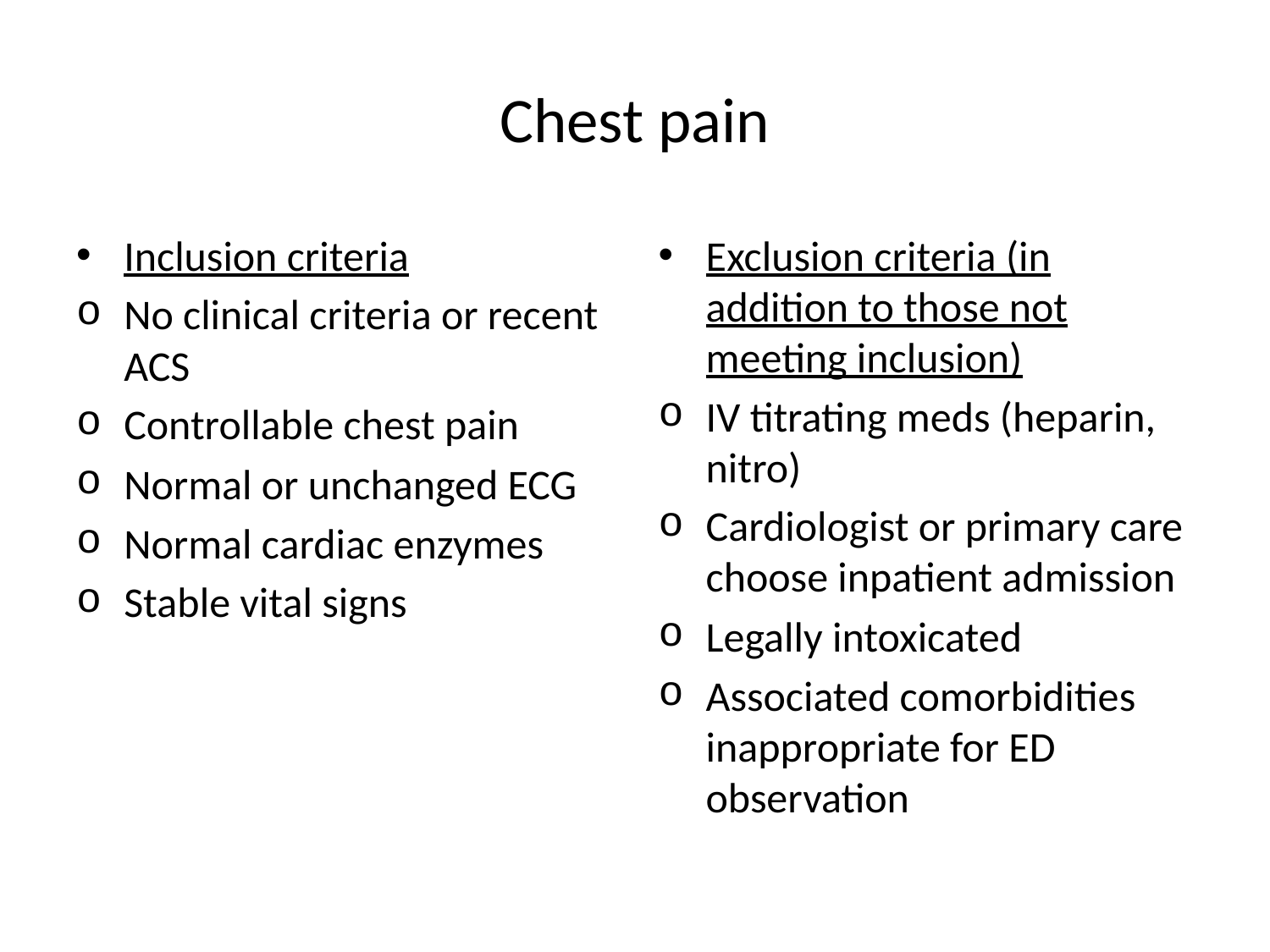

# Chest pain
Inclusion criteria
No clinical criteria or recent ACS
Controllable chest pain
Normal or unchanged ECG
Normal cardiac enzymes
Stable vital signs
Exclusion criteria (in addition to those not meeting inclusion)
IV titrating meds (heparin, nitro)
Cardiologist or primary care choose inpatient admission
Legally intoxicated
Associated comorbidities inappropriate for ED observation

## Slide 9
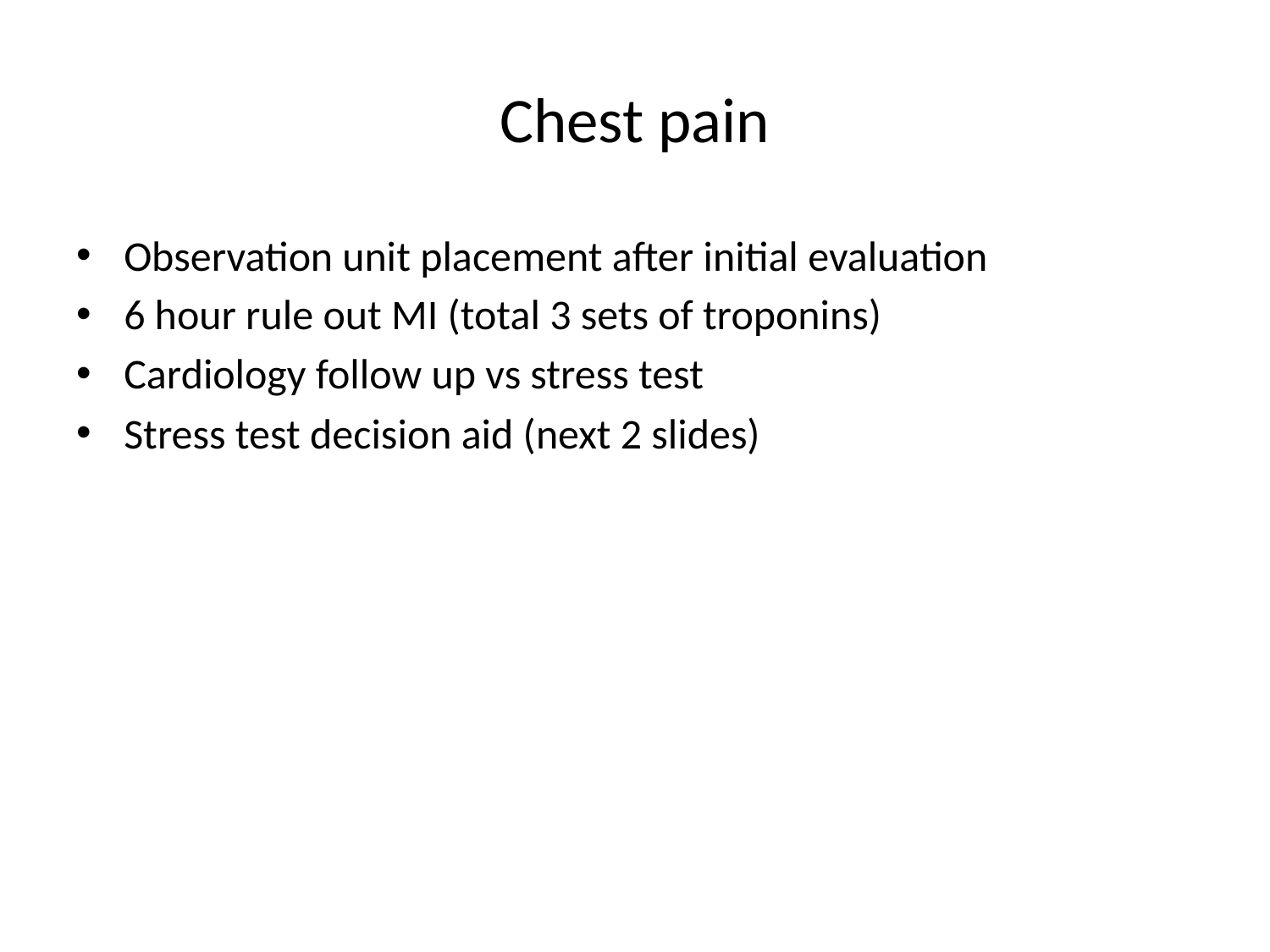

# Chest pain
Observation unit placement after initial evaluation
6 hour rule out MI (total 3 sets of troponins)
Cardiology follow up vs stress test
Stress test decision aid (next 2 slides)

## Slide 10
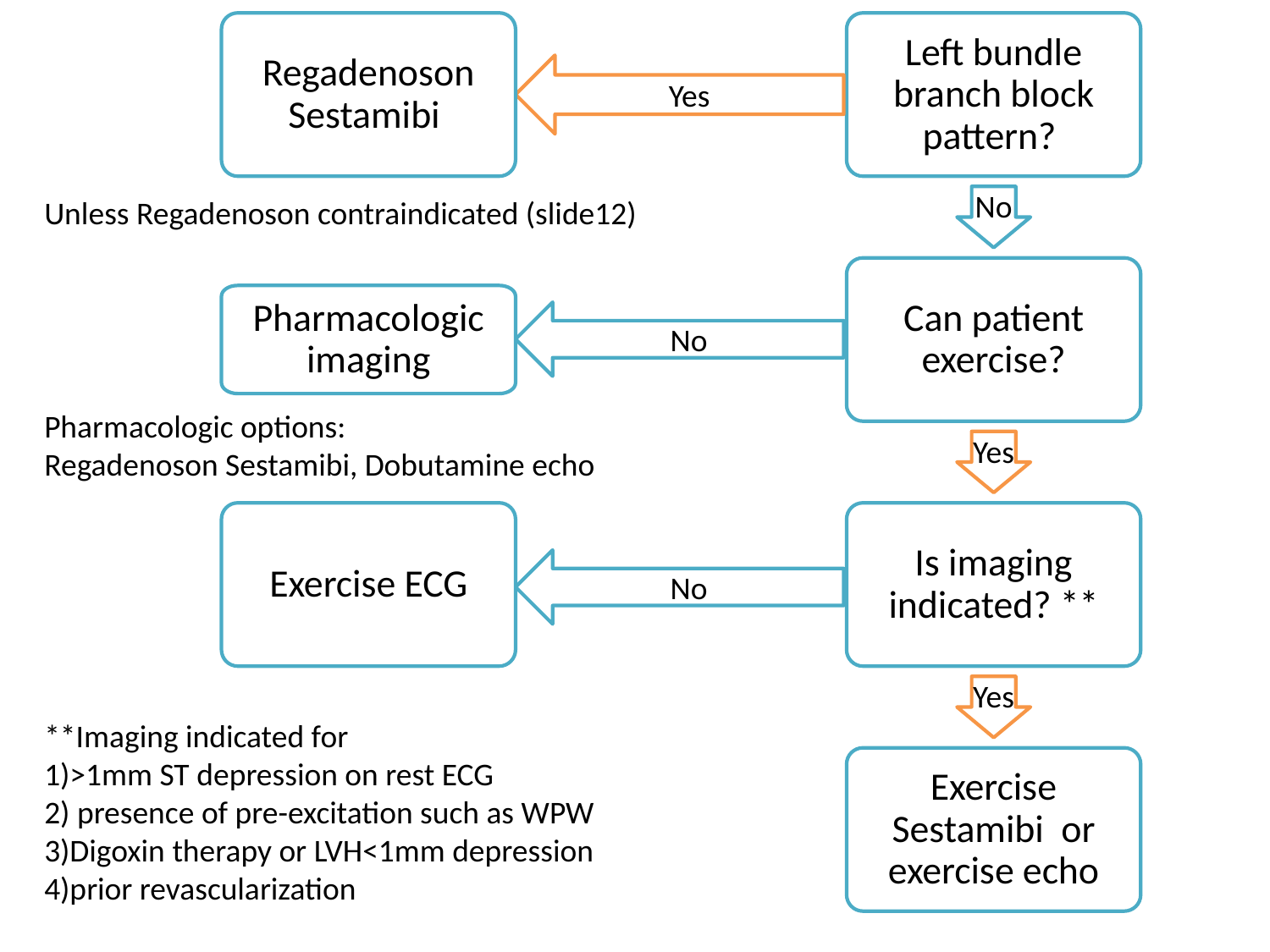

Regadenoson Sestamibi
Left bundle branch block pattern?
No
Can patient exercise?
Yes
Is imaging indicated? **
Yes
Exercise Sestamibi or exercise echo
Yes
Unless Regadenoson contraindicated (slide12)
Pharmacologic imaging
No
Pharmacologic options:
Regadenoson Sestamibi, Dobutamine echo
Exercise ECG
No
**Imaging indicated for
1)>1mm ST depression on rest ECG
2) presence of pre-excitation such as WPW 3)Digoxin therapy or LVH<1mm depression 4)prior revascularization

## Slide 11
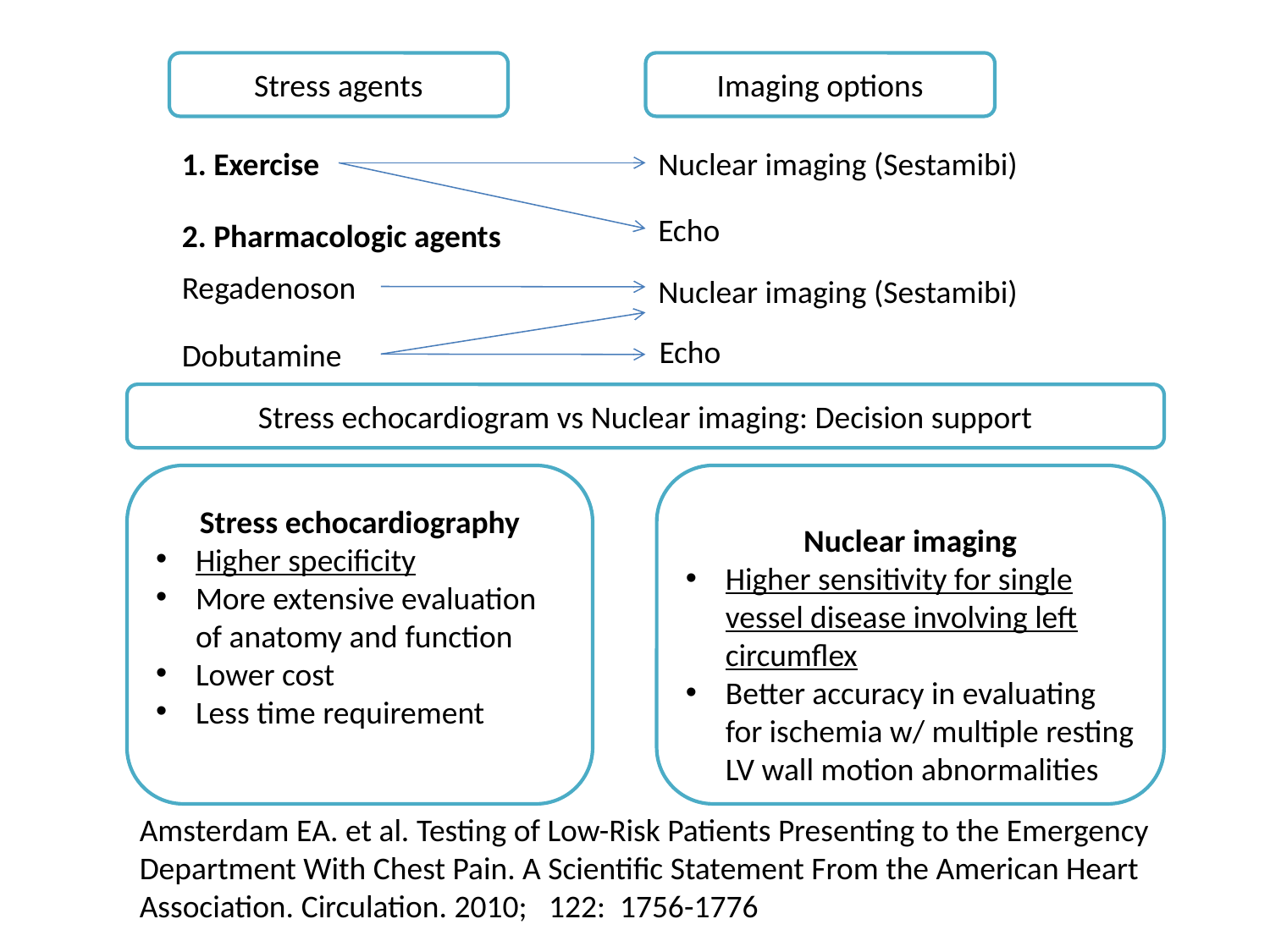

Stress agents
Imaging options
1. Exercise
Nuclear imaging (Sestamibi)
Echo
2. Pharmacologic agents
Regadenoson
Nuclear imaging (Sestamibi)
Echo
Dobutamine
Stress echocardiogram vs Nuclear imaging: Decision support
Stress echocardiography
Higher specificity
More extensive evaluation of anatomy and function
Lower cost
Less time requirement
Nuclear imaging
Higher sensitivity for single vessel disease involving left circumflex
Better accuracy in evaluating for ischemia w/ multiple resting LV wall motion abnormalities
Amsterdam EA. et al. Testing of Low-Risk Patients Presenting to the Emergency Department With Chest Pain. A Scientific Statement From the American Heart Association. Circulation. 2010; 122: 1756-1776

## Slide 12
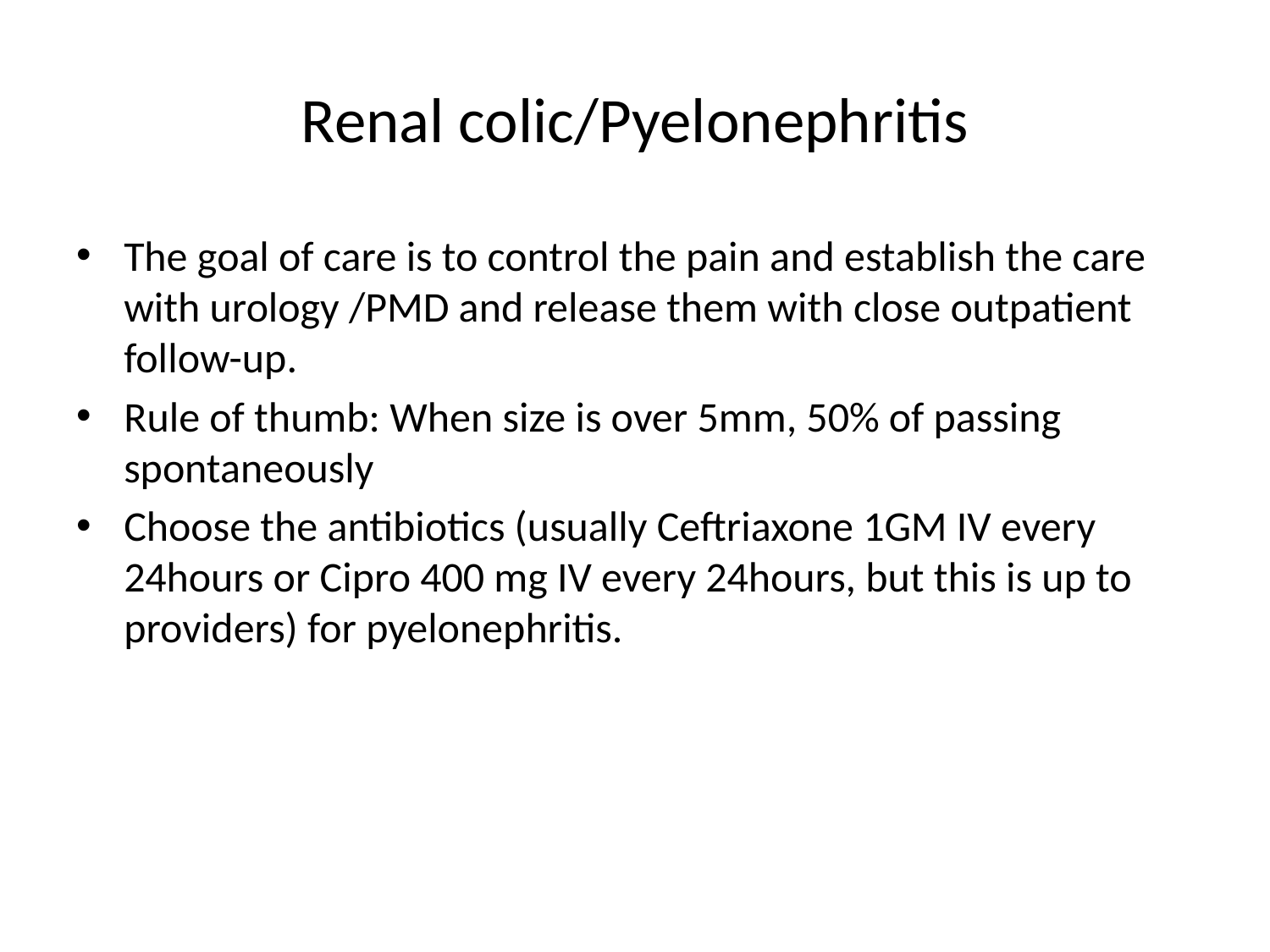

# Renal colic/Pyelonephritis
The goal of care is to control the pain and establish the care with urology /PMD and release them with close outpatient follow-up.
Rule of thumb: When size is over 5mm, 50% of passing spontaneously
Choose the antibiotics (usually Ceftriaxone 1GM IV every 24hours or Cipro 400 mg IV every 24hours, but this is up to providers) for pyelonephritis.

## Slide 13
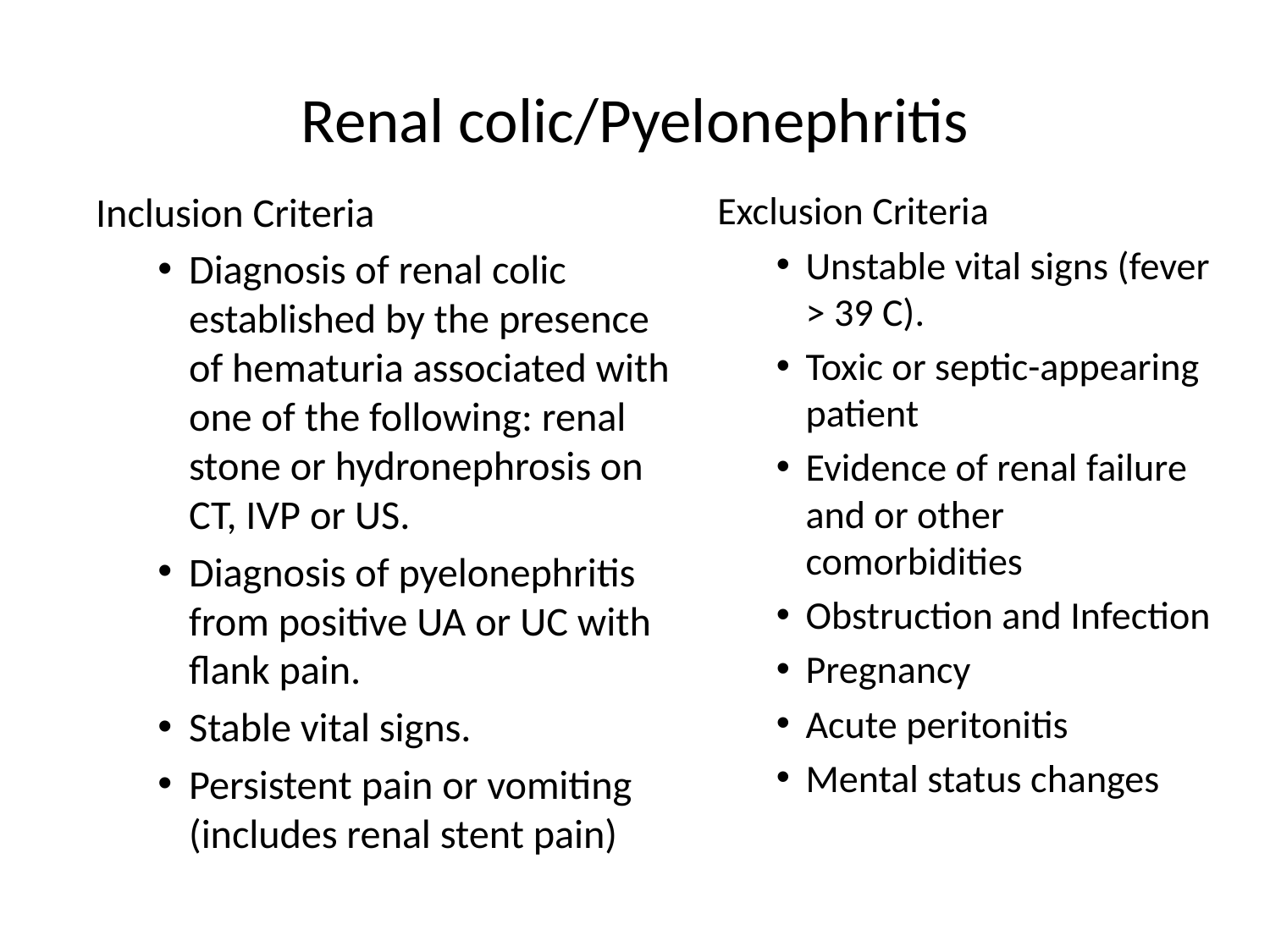

# Renal colic/Pyelonephritis
Inclusion Criteria
Diagnosis of renal colic established by the presence of hematuria associated with one of the following: renal stone or hydronephrosis on CT, IVP or US.
Diagnosis of pyelonephritis from positive UA or UC with flank pain.
Stable vital signs.
Persistent pain or vomiting (includes renal stent pain)
Exclusion Criteria
Unstable vital signs (fever > 39 C).
Toxic or septic-appearing patient
Evidence of renal failure and or other comorbidities
Obstruction and Infection
Pregnancy
Acute peritonitis
Mental status changes

## Slide 14
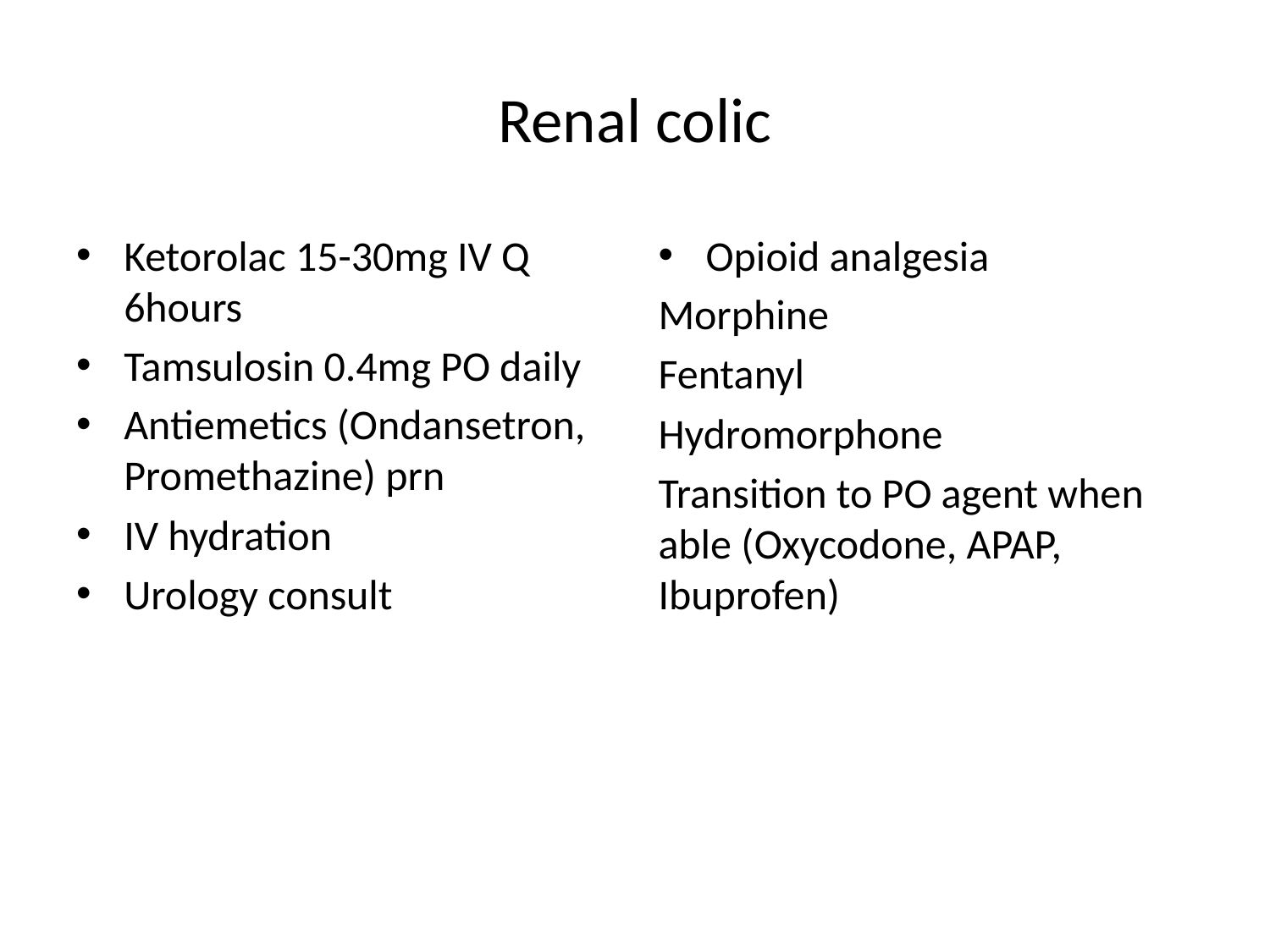

# Renal colic
Ketorolac 15-30mg IV Q 6hours
Tamsulosin 0.4mg PO daily
Antiemetics (Ondansetron, Promethazine) prn
IV hydration
Urology consult
Opioid analgesia
Morphine
Fentanyl
Hydromorphone
Transition to PO agent when able (Oxycodone, APAP, Ibuprofen)

## Slide 15
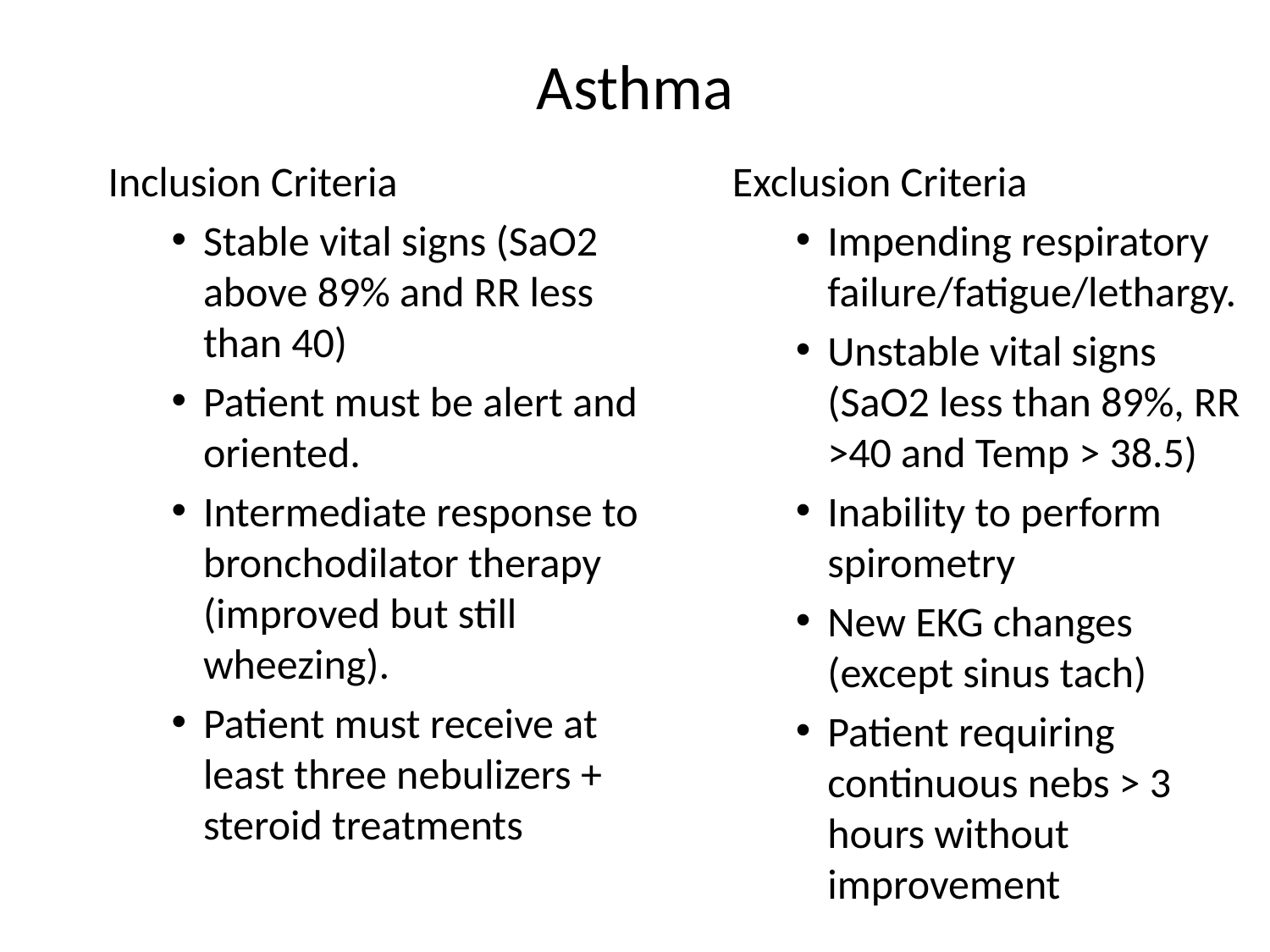

# Asthma
Inclusion Criteria
Stable vital signs (SaO2 above 89% and RR less than 40)
Patient must be alert and oriented.
Intermediate response to bronchodilator therapy (improved but still wheezing).
Patient must receive at least three nebulizers + steroid treatments
Exclusion Criteria
Impending respiratory failure/fatigue/lethargy.
Unstable vital signs (SaO2 less than 89%, RR >40 and Temp > 38.5)
Inability to perform spirometry
New EKG changes (except sinus tach)
Patient requiring continuous nebs > 3 hours without improvement

## Slide 16
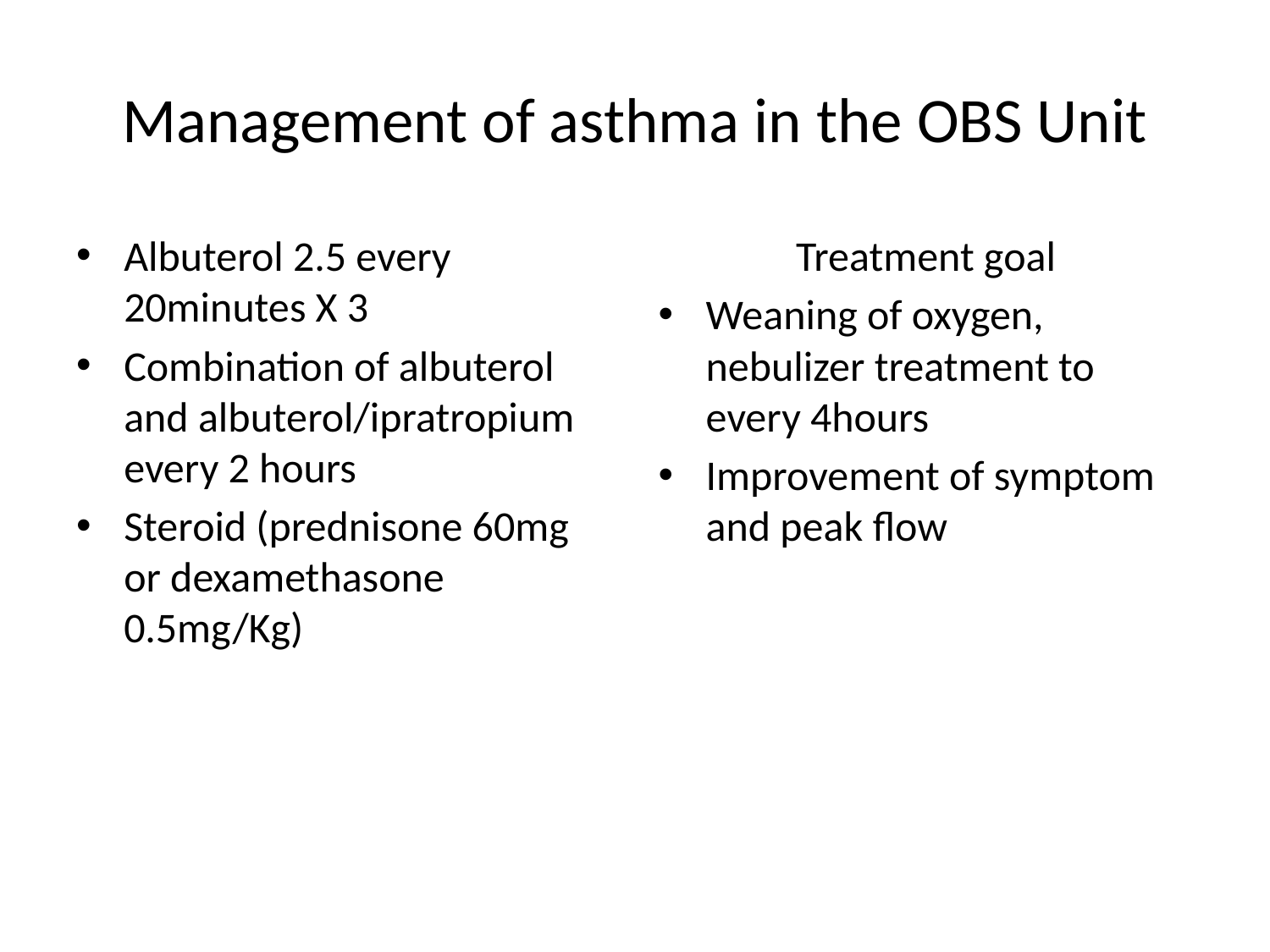

# Management of asthma in the OBS Unit
Albuterol 2.5 every 20minutes X 3
Combination of albuterol and albuterol/ipratropium every 2 hours
Steroid (prednisone 60mg or dexamethasone 0.5mg/Kg)
Treatment goal
Weaning of oxygen, nebulizer treatment to every 4hours
Improvement of symptom and peak flow

## Slide 17
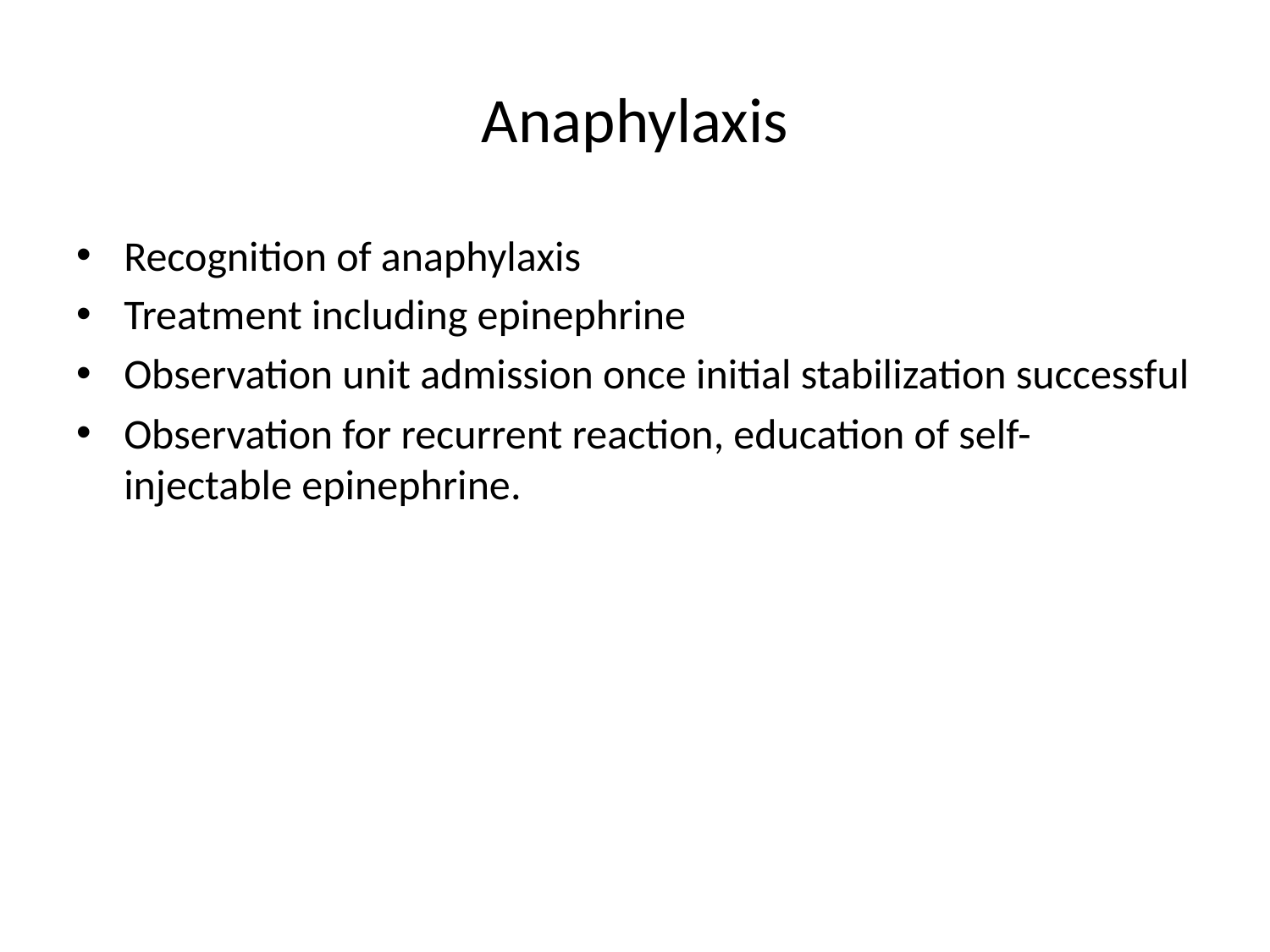

# Anaphylaxis
Recognition of anaphylaxis
Treatment including epinephrine
Observation unit admission once initial stabilization successful
Observation for recurrent reaction, education of self-injectable epinephrine.

## Slide 18
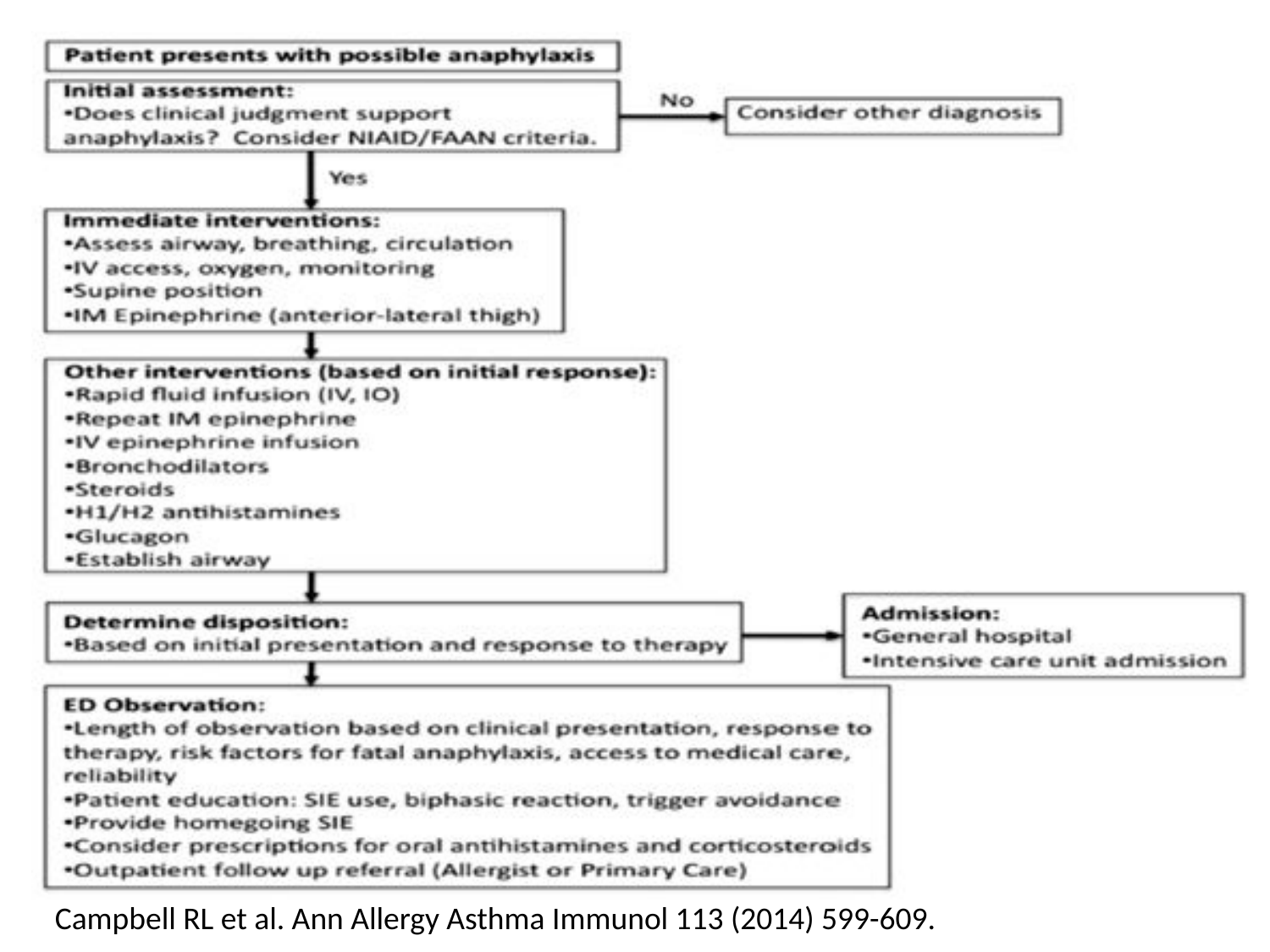

Campbell RL et al. Ann Allergy Asthma Immunol 113 (2014) 599-609.

## Slide 19
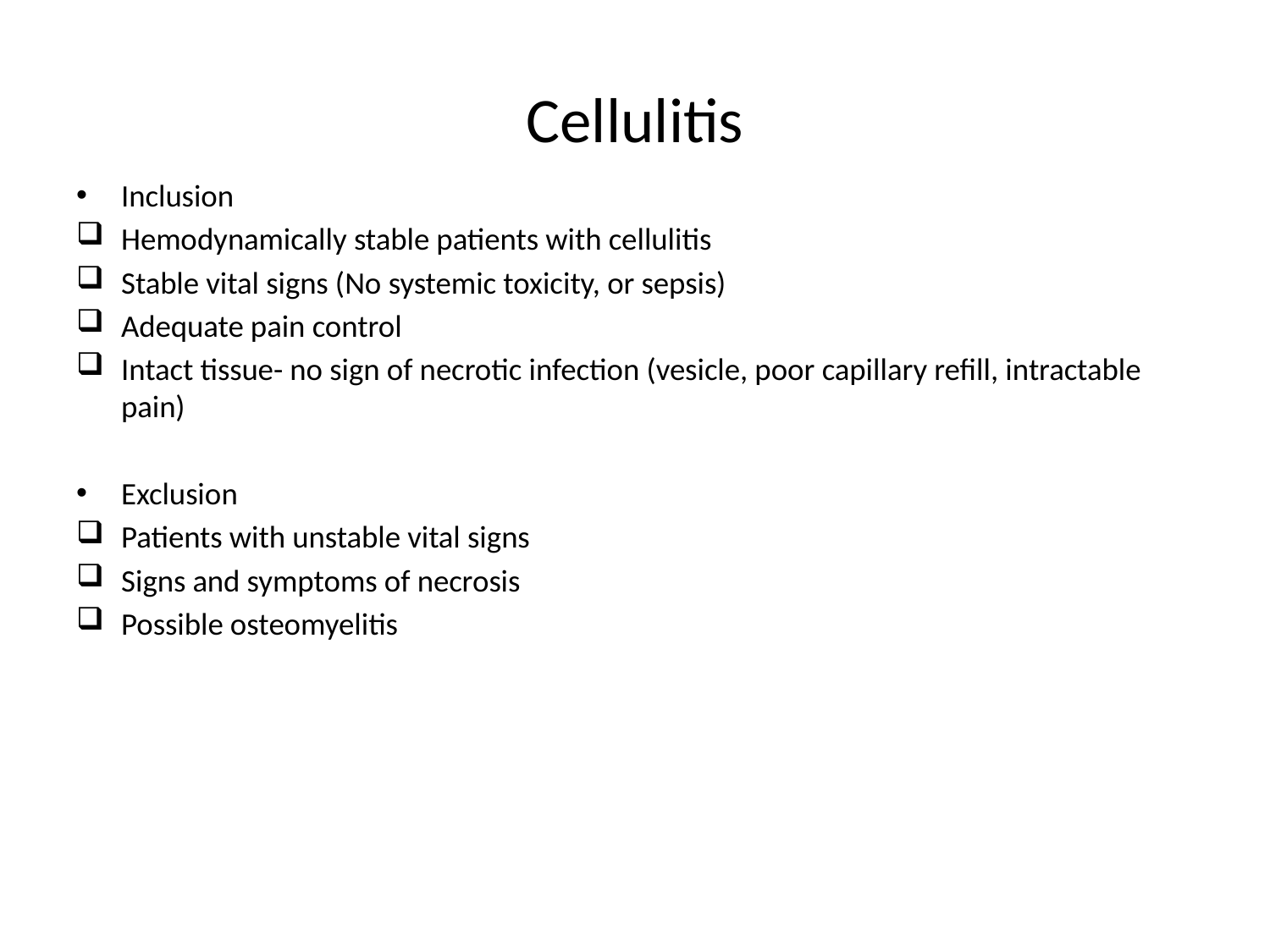

# Cellulitis
Inclusion
Hemodynamically stable patients with cellulitis
Stable vital signs (No systemic toxicity, or sepsis)
Adequate pain control
Intact tissue- no sign of necrotic infection (vesicle, poor capillary refill, intractable pain)
Exclusion
Patients with unstable vital signs
Signs and symptoms of necrosis
Possible osteomyelitis

## Slide 20
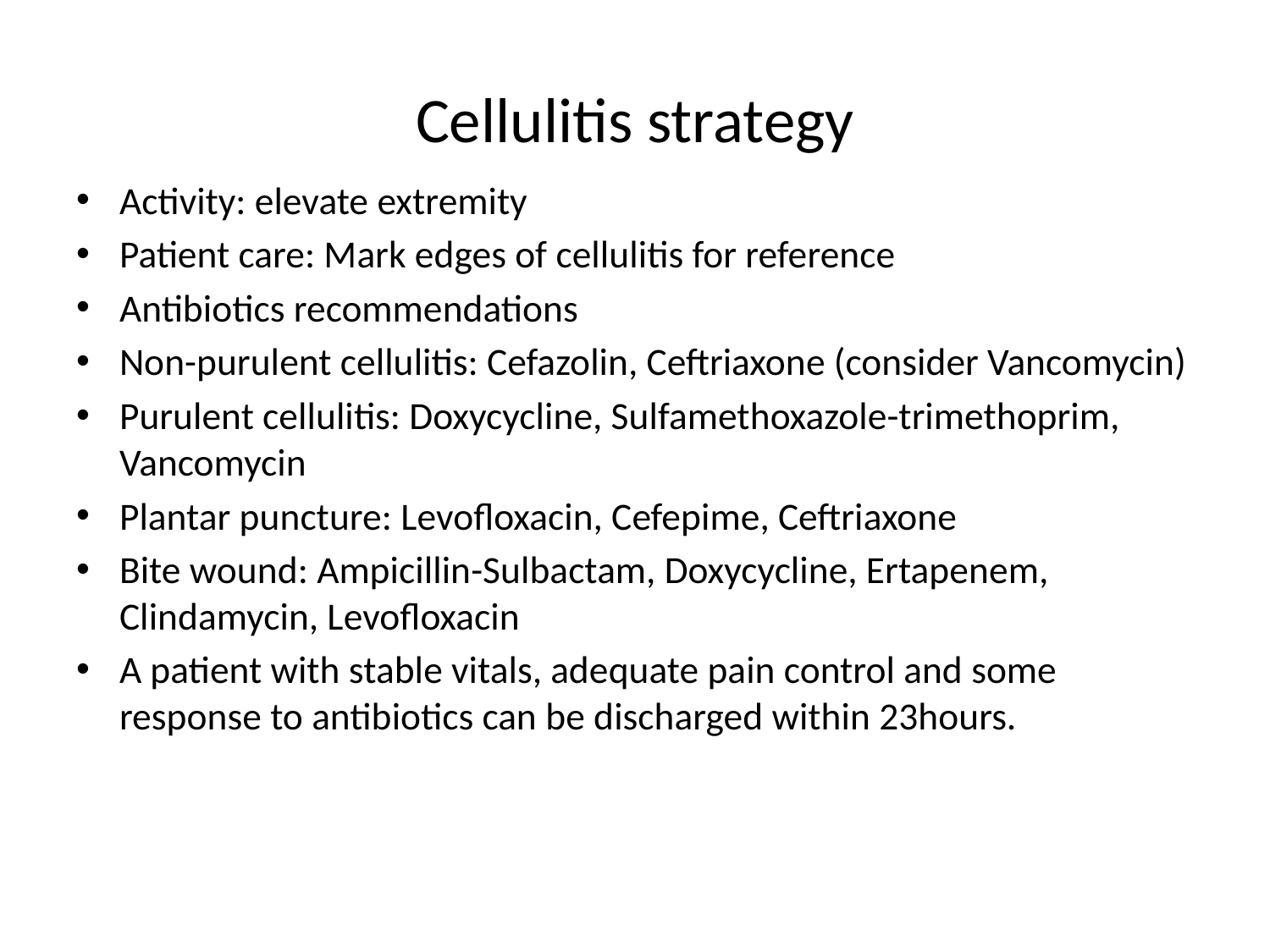

# Cellulitis strategy
Activity: elevate extremity
Patient care: Mark edges of cellulitis for reference
Antibiotics recommendations
Non-purulent cellulitis: Cefazolin, Ceftriaxone (consider Vancomycin)
Purulent cellulitis: Doxycycline, Sulfamethoxazole-trimethoprim, Vancomycin
Plantar puncture: Levofloxacin, Cefepime, Ceftriaxone
Bite wound: Ampicillin-Sulbactam, Doxycycline, Ertapenem, Clindamycin, Levofloxacin
A patient with stable vitals, adequate pain control and some response to antibiotics can be discharged within 23hours.

## Slide 21
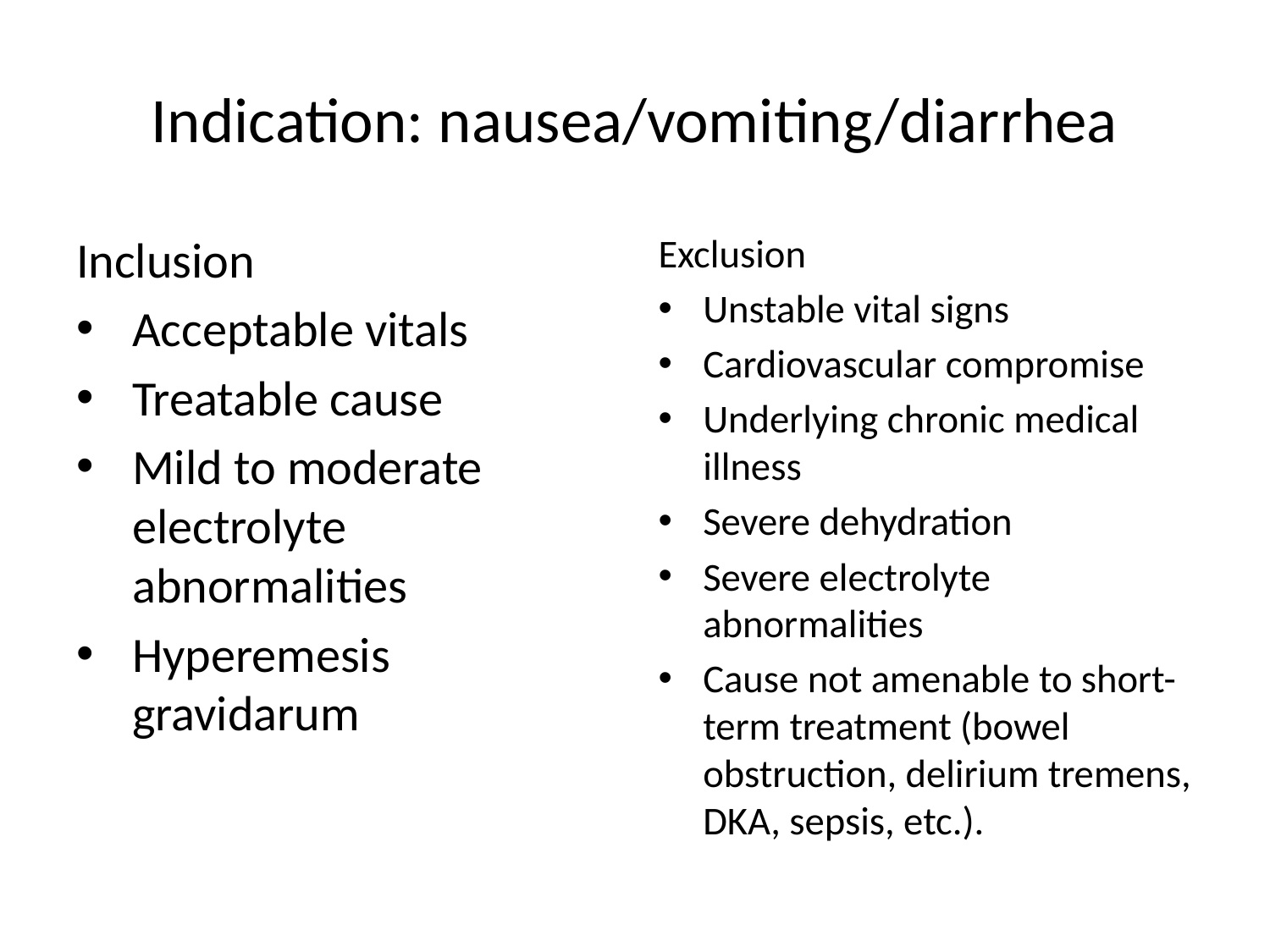

# Indication: nausea/vomiting/diarrhea
Inclusion
Acceptable vitals
Treatable cause
Mild to moderate electrolyte abnormalities
Hyperemesis gravidarum
Exclusion
Unstable vital signs
Cardiovascular compromise
Underlying chronic medical illness
Severe dehydration
Severe electrolyte abnormalities
Cause not amenable to short-term treatment (bowel obstruction, delirium tremens, DKA, sepsis, etc.).

## Slide 22
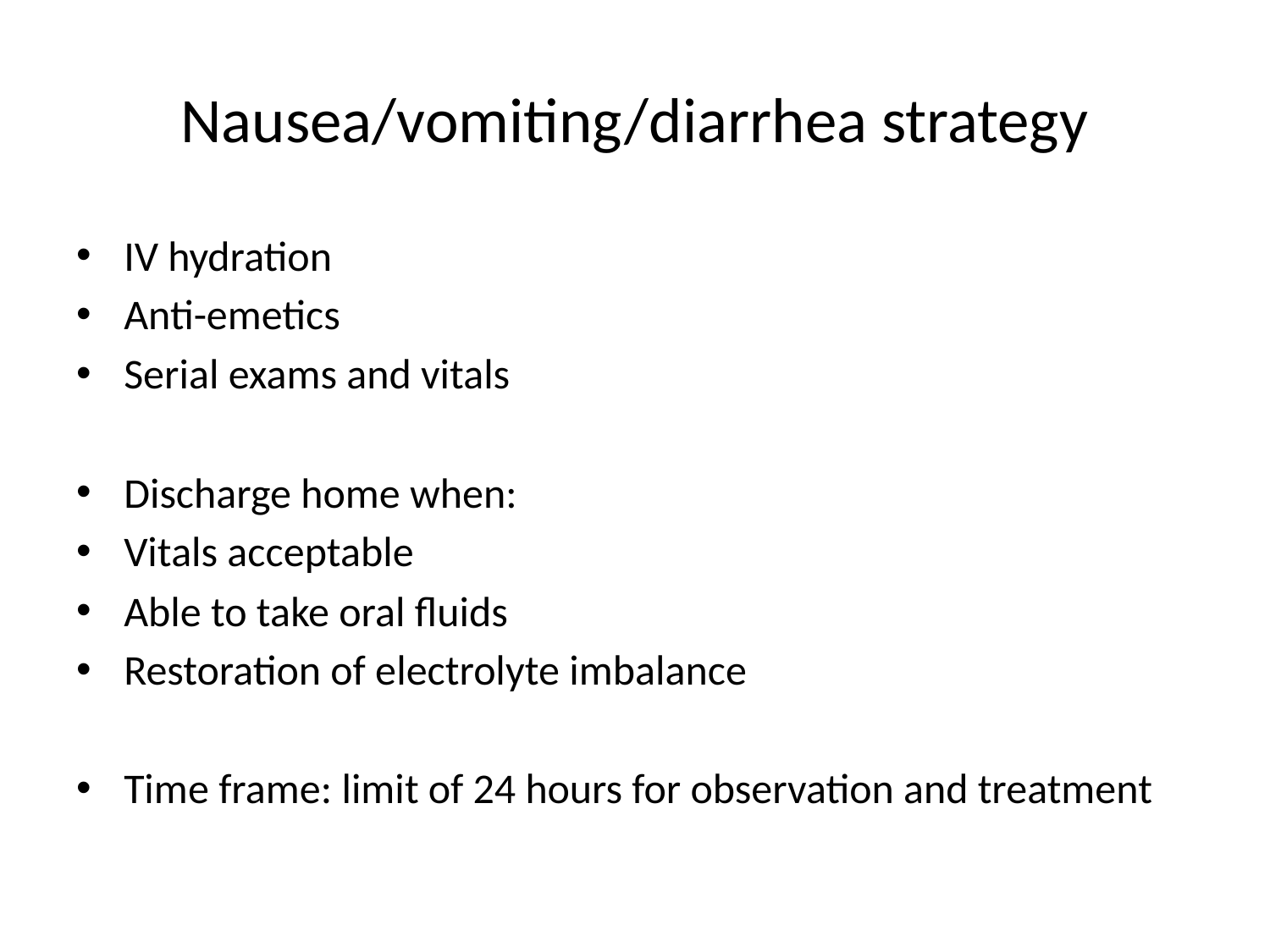

# Nausea/vomiting/diarrhea strategy
IV hydration
Anti-emetics
Serial exams and vitals
Discharge home when:
Vitals acceptable
Able to take oral fluids
Restoration of electrolyte imbalance
Time frame: limit of 24 hours for observation and treatment

## Slide 23
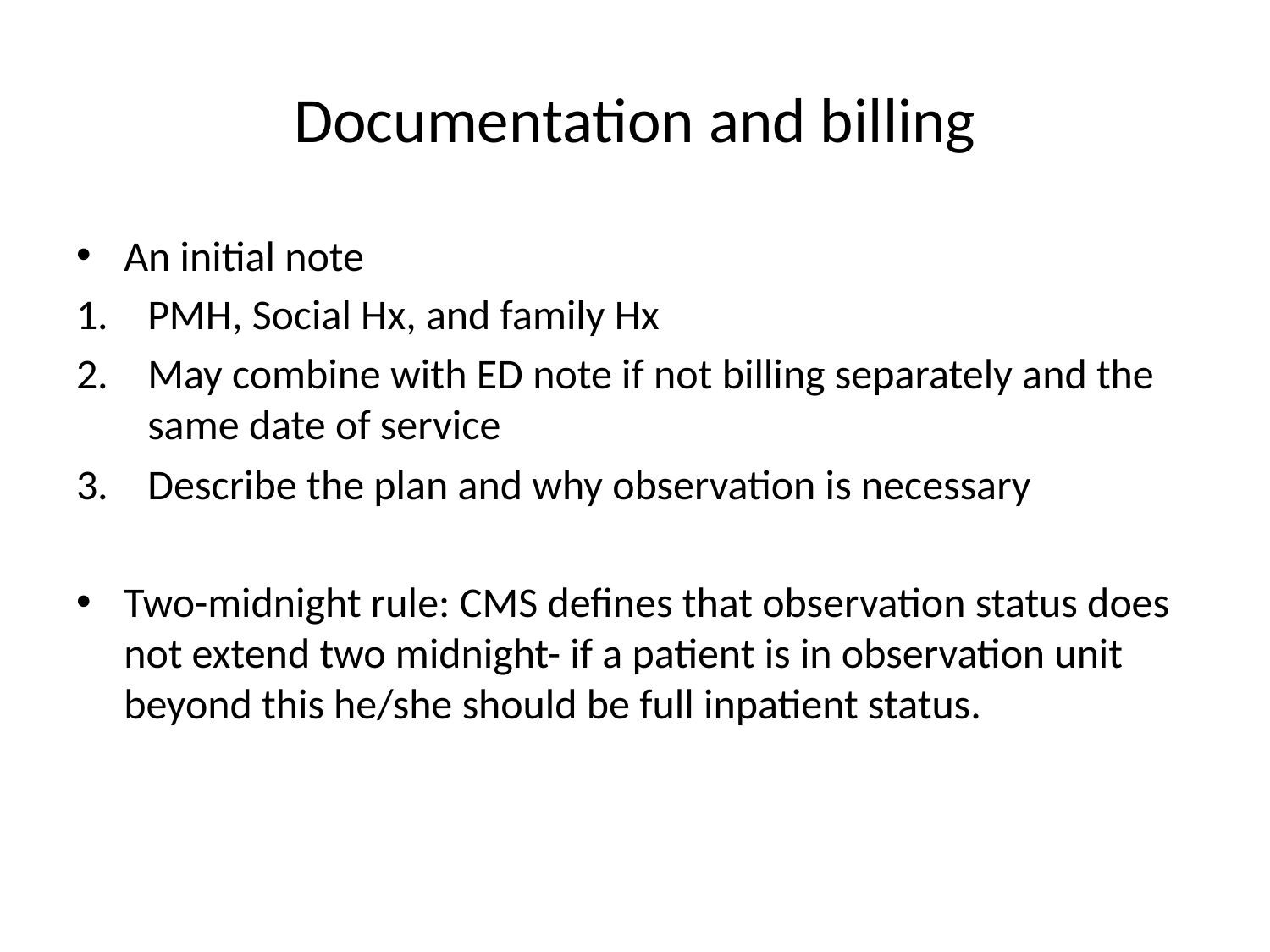

# Documentation and billing
An initial note
PMH, Social Hx, and family Hx
May combine with ED note if not billing separately and the same date of service
Describe the plan and why observation is necessary
Two-midnight rule: CMS defines that observation status does not extend two midnight- if a patient is in observation unit beyond this he/she should be full inpatient status.

## Slide 24
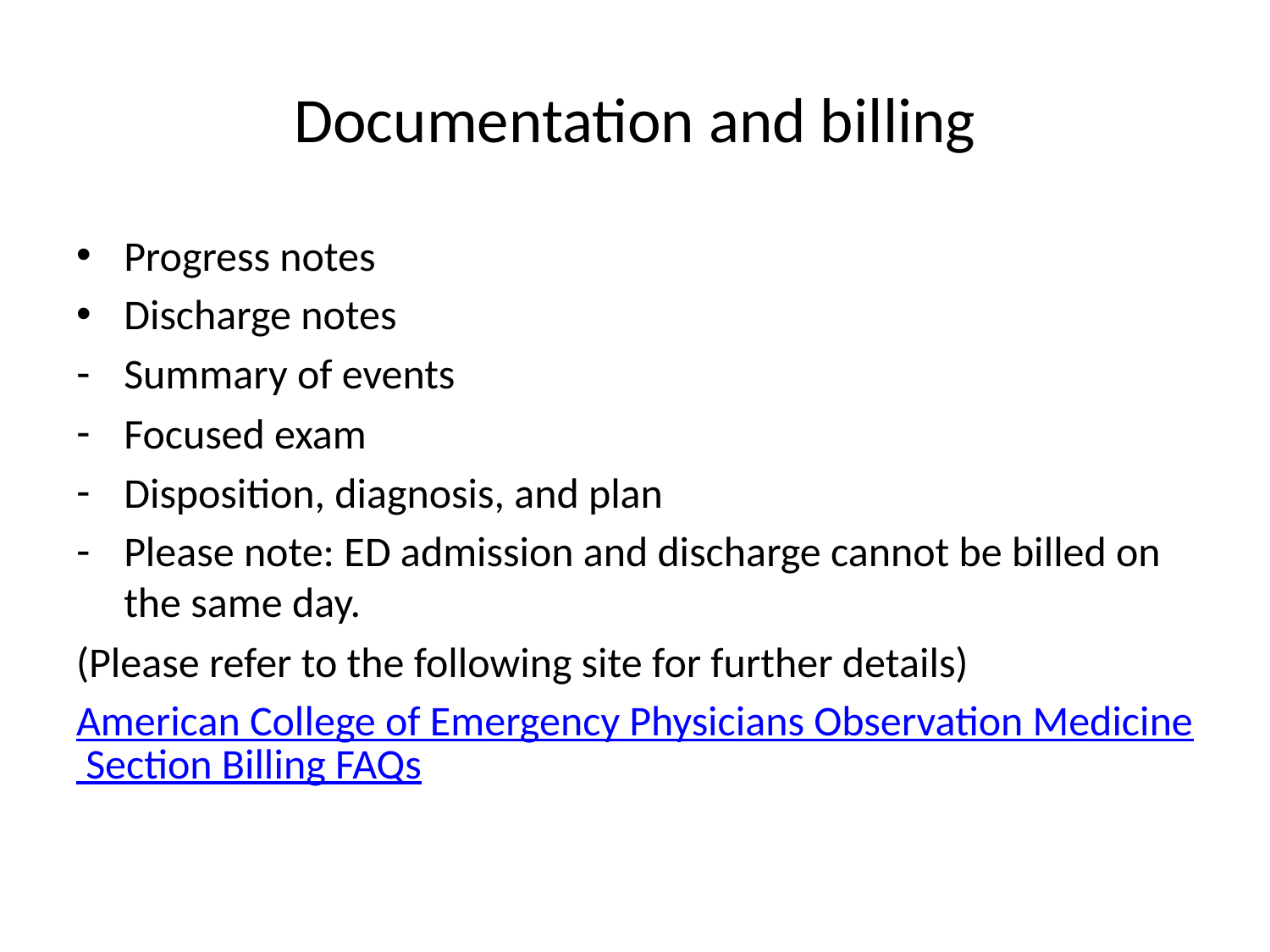

# Documentation and billing
Progress notes
Discharge notes
Summary of events
Focused exam
Disposition, diagnosis, and plan
Please note: ED admission and discharge cannot be billed on the same day.
(Please refer to the following site for further details)
American College of Emergency Physicians Observation Medicine Section Billing FAQs

## Slide 25
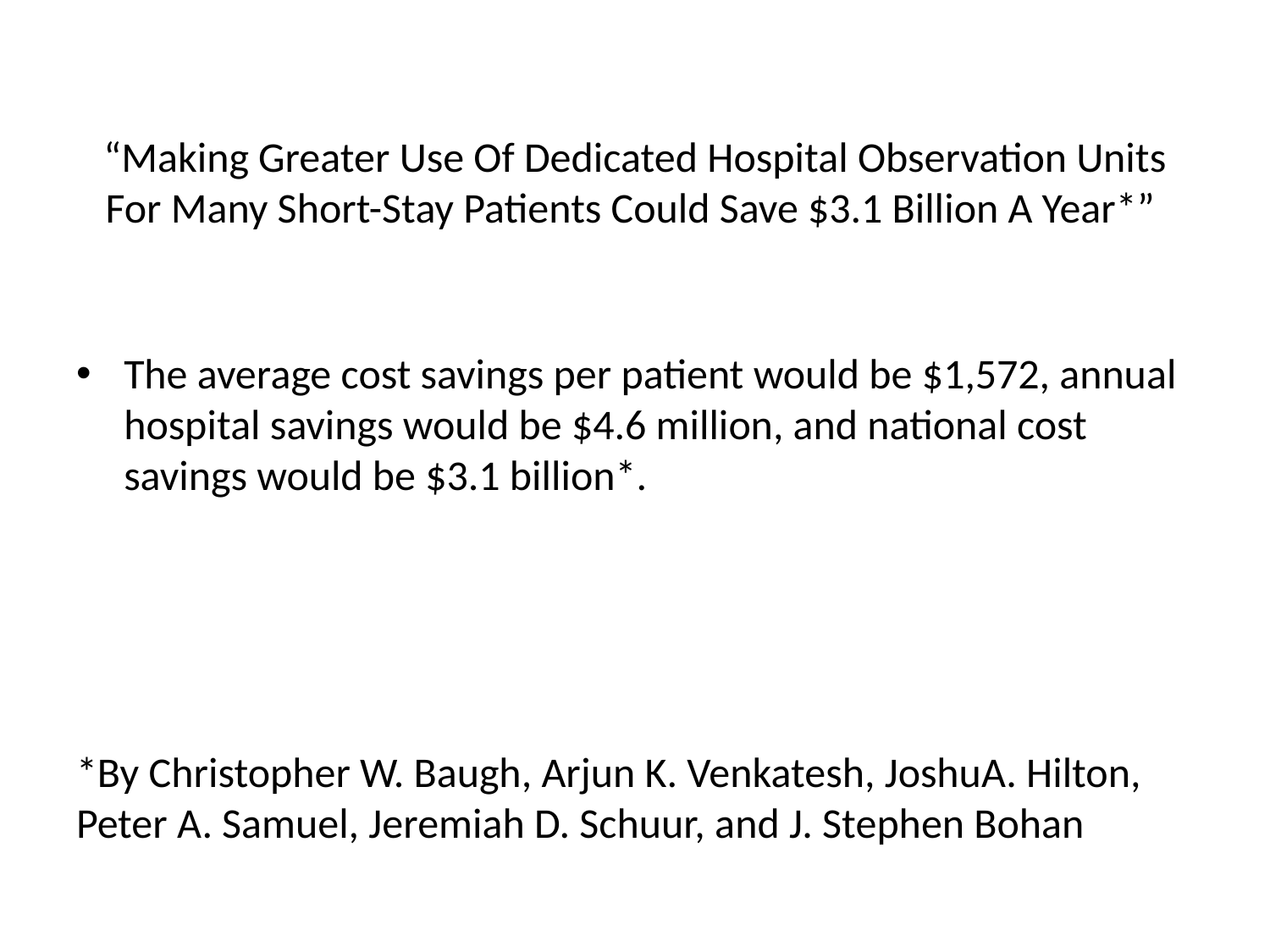

# “Making Greater Use Of Dedicated Hospital Observation Units For Many Short-Stay Patients Could Save $3.1 Billion A Year*”
The average cost savings per patient would be $1,572, annual hospital savings would be $4.6 million, and national cost savings would be $3.1 billion*.
*By Christopher W. Baugh, Arjun K. Venkatesh, JoshuA. Hilton, Peter A. Samuel, Jeremiah D. Schuur, and J. Stephen Bohan

## Slide 26
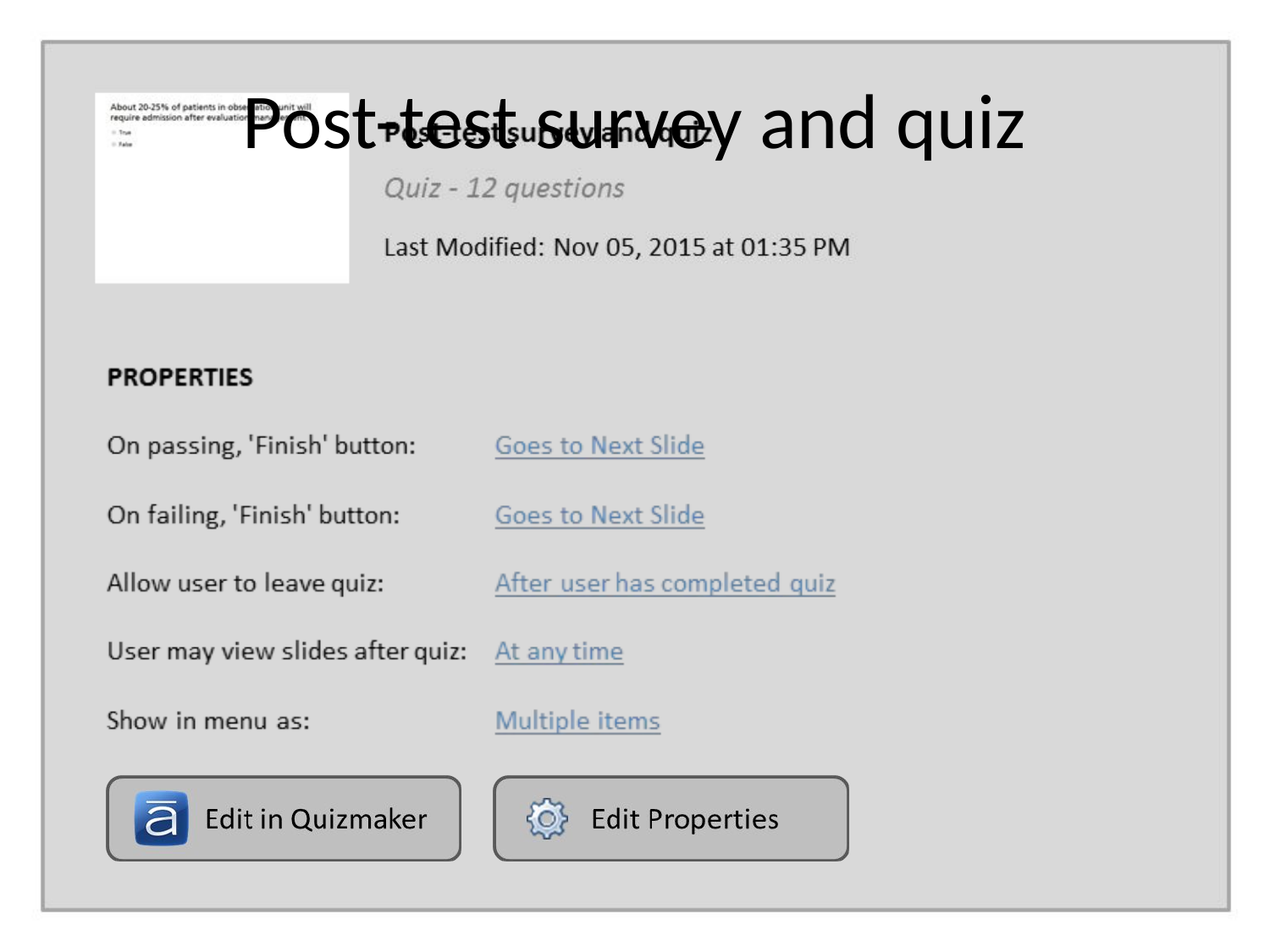

# Post-test survey and quiz

## Slide 27
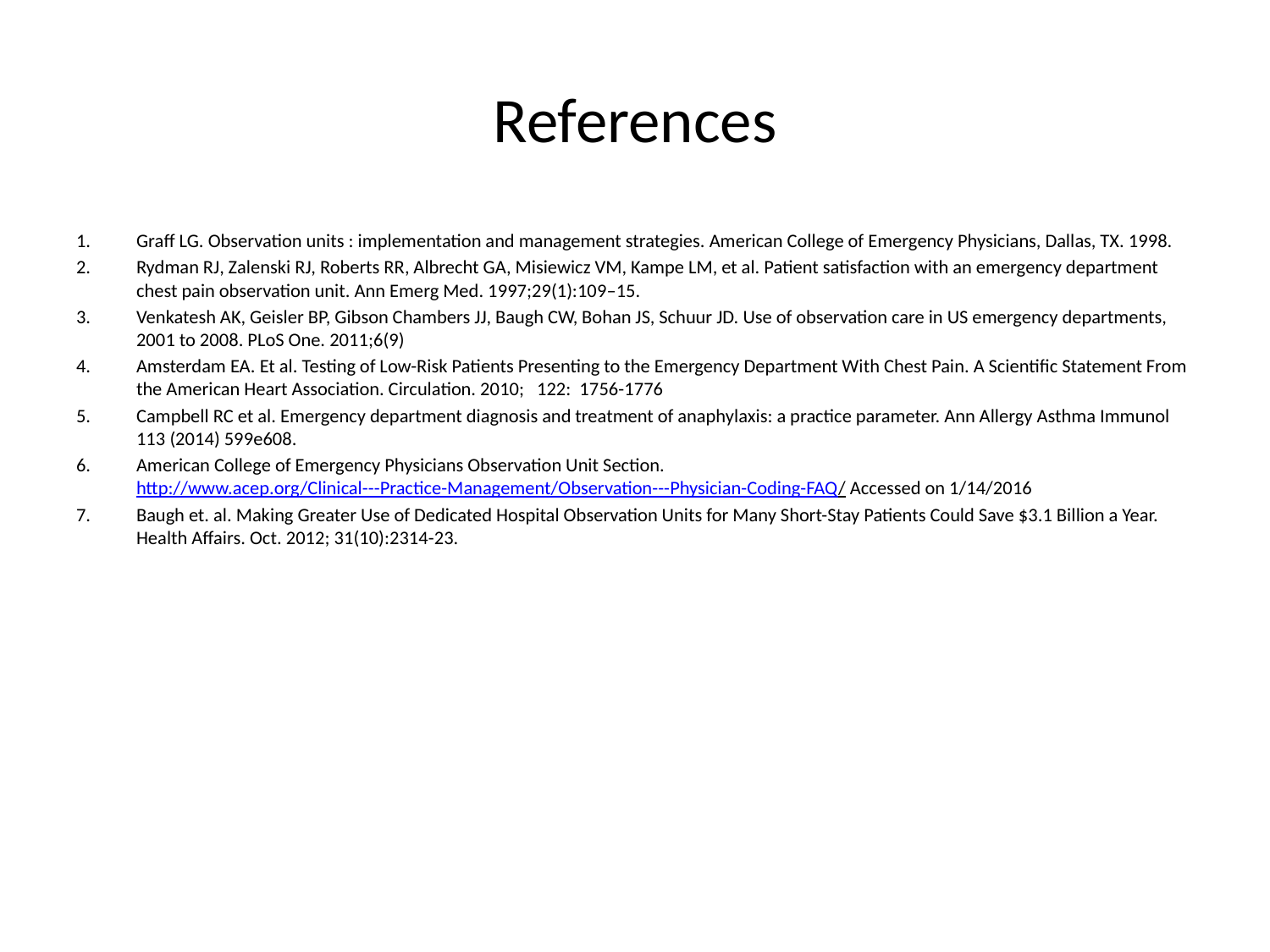

# References
Graff LG. Observation units : implementation and management strategies. American College of Emergency Physicians, Dallas, TX. 1998.
Rydman RJ, Zalenski RJ, Roberts RR, Albrecht GA, Misiewicz VM, Kampe LM, et al. Patient satisfaction with an emergency department chest pain observation unit. Ann Emerg Med. 1997;29(1):109–15.
Venkatesh AK, Geisler BP, Gibson Chambers JJ, Baugh CW, Bohan JS, Schuur JD. Use of observation care in US emergency departments, 2001 to 2008. PLoS One. 2011;6(9)
Amsterdam EA. Et al. Testing of Low-Risk Patients Presenting to the Emergency Department With Chest Pain. A Scientific Statement From the American Heart Association. Circulation. 2010; 122: 1756-1776
Campbell RC et al. Emergency department diagnosis and treatment of anaphylaxis: a practice parameter. Ann Allergy Asthma Immunol 113 (2014) 599e608.
American College of Emergency Physicians Observation Unit Section. http://www.acep.org/Clinical---Practice-Management/Observation---Physician-Coding-FAQ/ Accessed on 1/14/2016
Baugh et. al. Making Greater Use of Dedicated Hospital Observation Units for Many Short-Stay Patients Could Save $3.1 Billion a Year. Health Affairs. Oct. 2012; 31(10):2314-23.
