# Supplementary figures and images for: Online Module to Improve Emergency Department Observation Unit Practice
Source: MedEdPORTAL. 2016 Jul 8;12:10423. doi: 10.15766/mep_2374-8265.10423 (PMC6464449; doi:10.15766/mep_2374-8265.10423)

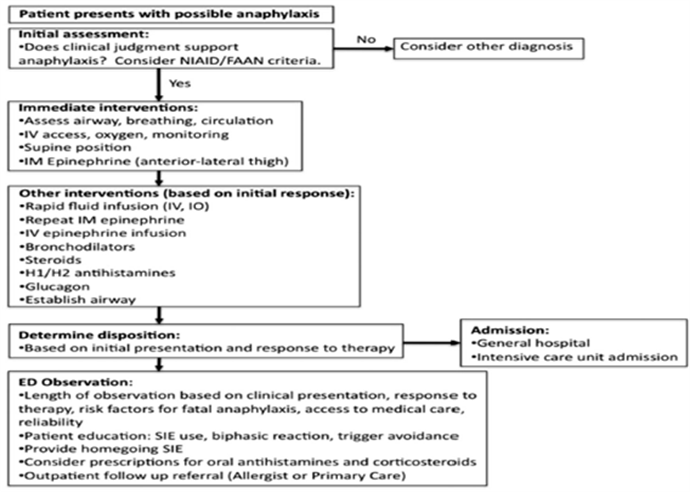

Supplement: Supplementary file 1 — A. Introducing Observation Medicine for Emergency Medicine Physicians Articulate folder B. Introducing Observation Medicine for Emergency Medicine Physicians PowerPoint.pptx C. Articulate Presentation Instructions.txt [file mep-12-10423-s001.zip › A. Introducing Observation Medicine for Emergency Medicine Physicians Articulate/mobile/6ddNWoa1uSc_6pOWyCKJnvM_80_DX1136_DY1136_CX690_CY492.png]

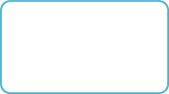

Supplement: Supplementary file 1 — A. Introducing Observation Medicine for Emergency Medicine Physicians Articulate folder B. Introducing Observation Medicine for Emergency Medicine Physicians PowerPoint.pptx C. Articulate Presentation Instructions.txt [file mep-12-10423-s001.zip › A. Introducing Observation Medicine for Emergency Medicine Physicians Articulate/mobile/6ddNWoa1uSc_sl264shp14_80_DX338_DY338_CX169_CY94.png]

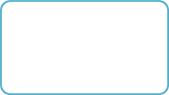

Supplement: Supplementary file 1 — A. Introducing Observation Medicine for Emergency Medicine Physicians Articulate folder B. Introducing Observation Medicine for Emergency Medicine Physicians PowerPoint.pptx C. Articulate Presentation Instructions.txt [file mep-12-10423-s001.zip › A. Introducing Observation Medicine for Emergency Medicine Physicians Articulate/mobile/6ddNWoa1uSc_sl264shp17_80_DX338_DY338_CX169_CY95.png]

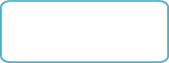

Supplement: Supplementary file 1 — A. Introducing Observation Medicine for Emergency Medicine Physicians Articulate folder B. Introducing Observation Medicine for Emergency Medicine Physicians PowerPoint.pptx C. Articulate Presentation Instructions.txt [file mep-12-10423-s001.zip › A. Introducing Observation Medicine for Emergency Medicine Physicians Articulate/mobile/6ddNWoa1uSc_sl264shp19_80_DX338_DY338_CX169_CY63.png]

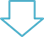

Supplement: Supplementary file 1 — A. Introducing Observation Medicine for Emergency Medicine Physicians Articulate folder B. Introducing Observation Medicine for Emergency Medicine Physicians PowerPoint.pptx C. Articulate Presentation Instructions.txt [file mep-12-10423-s001.zip › A. Introducing Observation Medicine for Emergency Medicine Physicians Articulate/mobile/6ddNWoa1uSc_sl264shp4idx2_80_DX88_DY88_CX44_CY37.png]

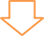

Supplement: Supplementary file 1 — A. Introducing Observation Medicine for Emergency Medicine Physicians Articulate folder B. Introducing Observation Medicine for Emergency Medicine Physicians PowerPoint.pptx C. Articulate Presentation Instructions.txt [file mep-12-10423-s001.zip › A. Introducing Observation Medicine for Emergency Medicine Physicians Articulate/mobile/6ddNWoa1uSc_sl264shp4idx4_80_DX88_DY88_CX44_CY36.png]

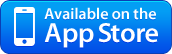

Supplement: Supplementary file 1 — A. Introducing Observation Medicine for Emergency Medicine Physicians Articulate folder B. Introducing Observation Medicine for Emergency Medicine Physicians PowerPoint.pptx C. Articulate Presentation Instructions.txt [file mep-12-10423-s001.zip › A. Introducing Observation Medicine for Emergency Medicine Physicians Articulate/mobile/app_store.png]

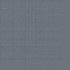

Supplement: Supplementary file 1 — A. Introducing Observation Medicine for Emergency Medicine Physicians Articulate folder B. Introducing Observation Medicine for Emergency Medicine Physicians PowerPoint.pptx C. Articulate Presentation Instructions.txt [file mep-12-10423-s001.zip › A. Introducing Observation Medicine for Emergency Medicine Physicians Articulate/mobile/linen_background.jpg]

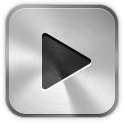

Supplement: Supplementary file 1 — A. Introducing Observation Medicine for Emergency Medicine Physicians Articulate folder B. Introducing Observation Medicine for Emergency Medicine Physicians PowerPoint.pptx C. Articulate Presentation Instructions.txt [file mep-12-10423-s001.zip › A. Introducing Observation Medicine for Emergency Medicine Physicians Articulate/mobile/mobile_icon.png]

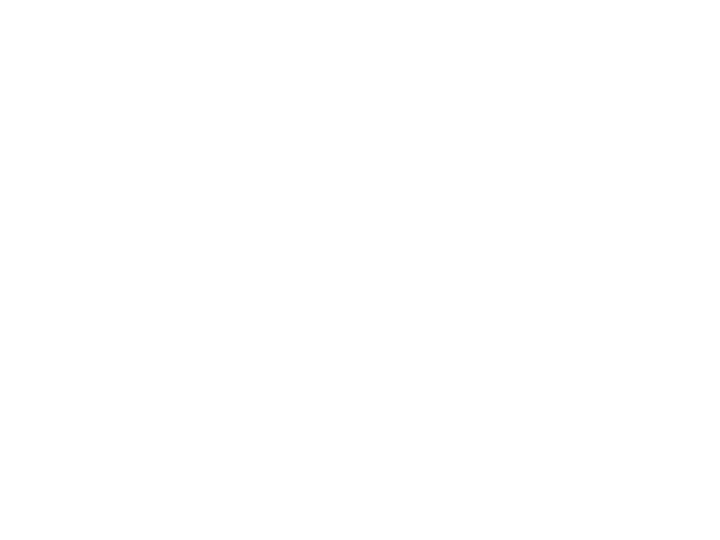

Supplement: Supplementary file 1 — A. Introducing Observation Medicine for Emergency Medicine Physicians Articulate folder B. Introducing Observation Medicine for Emergency Medicine Physicians PowerPoint.pptx C. Articulate Presentation Instructions.txt [file mep-12-10423-s001.zip › A. Introducing Observation Medicine for Emergency Medicine Physicians Articulate/mobile/Slide6ddNWoa1uSc_d1sm.jpg]

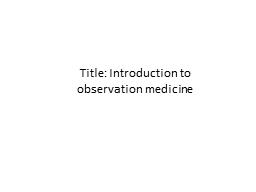

Supplement: Supplementary file 1 — A. Introducing Observation Medicine for Emergency Medicine Physicians Articulate folder B. Introducing Observation Medicine for Emergency Medicine Physicians PowerPoint.pptx C. Articulate Presentation Instructions.txt [file mep-12-10423-s001.zip › A. Introducing Observation Medicine for Emergency Medicine Physicians Articulate/presentation_content/thumbnail.jpg]
